# Genome-wide identification of CAD and CCoAOMT gene families in soybean and analysis of expression patterns under *Peronospora manshurica* (*P. manshurica*) infection

**DOI:** 10.3389/fpls.2026.1834683

**Published:** 2026-06-16

**Authors:** Biao Xu, Xiaodong Liu, Dawei Li, Guangxun Qi, Cuiping Yuan, Yumin Wang, Yingshan Dong, Hongkun Zhao

**Affiliations:** 1 Jilin Academy of Agricultural Sciences (Northeast Agricultural Research Center of China), Soybean Research Institute, Changchun, China; 2 Jilin Agricultural University, College of Agriculture, Changchun, China; 3 Yanbian University, College of Agriculture, Yanji, China

**Keywords:** caffeoyl-coenzyme A 3-O-methyltransferase, cinnamyl alcohol dehydrogenase, expression pattern analysis, *P. manshurica* infection, soybean

## Abstract

**Background:**

Cinnamyl alcohol dehydrogenase (CAD) and caffeoyl–coenzyme A 3-O-methyltransferase (CCoAOMT) are key enzymes in lignin biosynthesis, playing important roles in plant growth, development, and stress responses. However, the evolutionary relationships and functions of these two gene families in soybean remain poorly understood.

**Methods:**

In this study, a genome-wide identification of the CAD and CCoAOMT gene families in soybean was conducted to analyze their physicochemical properties, gene structures, conserved motifs, and chromosomal distributions. Their phylogenetic relationships, collinearity, promoter cis-elements, miRNA targeting, protein interaction networks, and tissue-specific expression patterns were investigated. The expression profiles under *P. manshurica* infection were validated using qRT-PCR.

**Results:**

A total of 41 *GmCAD* and 13 *GmCCoAOMT* genes were identified in the soybean genome. *GmCAD* proteins are predominantly hydrophobic and stable, while *GmCCoAOMT* proteins are mainly hydrophilic; both are localized in the cytoplasm. *GmCAD* genes are classified into three subgroups containing eight conserved motifs, whereas *GmCCoAOMT* genes fall into four subgroups with seven motifs. Members within the same subgroup exhibit highly similar gene structures and motif compositions. Promoter analysis revealed core elements (CAAT-box, TATA-box) along with light-, hormone-, and stress-responsive cis-elements. A total of 161 miRNAs were predicted to target 13 *GmCAD* genes and 39 miRNAs to target 13 *GmCCoAOMT* genes, mostly in a one-miRNA–one-target manner. Extensive protein–protein interactions were observed among *GmCAD* proteins, but none among *GmCCoAOMT* proteins or between the two families. All genes underwent purifying selection (Ka/Ks < 1) and showed closer evolutionary relationships with *Arabidopsis* than with rice. Both gene families exhibited distinct tissue-specific expression patterns. Based on expression profiling, *GmCAD14*, *GmCAD26*, *GmCAD28*, *GmCAD36*, *GmCCoAOMT1*, *GmCCoAOMT3*, *GmCCoAOMT4*, and *GmCCoAOMT5* are proposed as candidate resistance genes against *P. manshurica* infection.

**Conclusions:**

This study reveals the evolutionary characteristics and regulatory networks of the CAD and CCoAOMT gene families in soybean, laying a foundation for further research into their roles in disease resistance.

## 1 Introduction

Soybean (*Glycine max* (L.) Merr.) is a globally significant food and economic crop, its yield and quality directly affect food security and agricultural economic benefits (Wang et al., 2025).

However, soybeans are vulnerable to various pathogens during their growth process, causing significant yield loss. Its disease resistance is regulated by multiple interconnected physiological processes, among which secondary metabolite synthesis and accumulation play a central role. Lignin, an important aromatic polymer, accumulates rapidly under pathogen stress, forming a physical barrier that prevents pathogen spread and enhances disease resistance (Xun et al., 2022).

Lignin biosynthesis is a complex multi-step enzymatic reaction process involving two key pathways, namely: phenylpropanoid metabolic pathway and lignin monomer specific synthesis pathway, which is regulated by a series of structural genes and regulatory genes (Boerjan et al., 2003; Vanholme et al., 2010). CAD and CCoAOMT are two key rate-limiting enzymes in the lignin synthesis pathway, which directly determine the total amount, composition, and structural characteristics of lignin (Jun et al., 2017; Zhang et al., 2019).

CAD mainly catalyzes the last step of the reduction reaction in the lignin synthesis pathway, reducing cinnamaldehyde compounds (such as cinnamaldehyde, coumaraldehyde, coniferyl aldehyde, and sinapaldehyde) to cinnamyl alcohol compounds (such as cinnamyl alcohol, coumaranol, coniferyl alcohol, and sinapyl alcohol), and these cinnamyl alcohol compounds are the basic monomers that constitute lignin (such as guaiacyl lignin monomer and syringyl lignin monomer) (Mansell et al., 1974; Wyrambik and Grisebach, 1975). Studies have shown that the expression level and enzyme activity of CAD genes play an important role in the synthesis of lignin monomers and plant disease resistance—for example, in *Arabidopsis*, mutations in CAD-C and CAD-D genes significantly reduce plant resistance to pathogenic bacteria (such as the tomato bacteria spot pathogen), and this effect may interact with the salicylic acid signaling pathway (Tronchet et al., 2010). In upland cotton, silencing *GhCAD35*, *GhCAD45*, or *GhCAD43* inhibits salicylic acid biosynthesis and stem lignification, thereby significantly reducing plant resistance to *Verticillium dahliae* (Li et al., 2022).

CCoAOMT is mainly involved in the synthesis of guaiacyl lignin (G-lignin). Its core function is to catalyze the methylation of caffeoyl-CoA to produce feruloyl-CoA, which provides key intermediates for the synthesis of coniferyl alcohol (G-lignin precursor). Its activity directly determines the synthesis efficiency of G-lignin and ultimately affects the structural stability and chemical properties of lignin (Guo et al., 2001; Hoffmann et al., 2001)—for example, in sorghum, overexpression of *SbCCoAOMT* resulted in reduced reproduction of sugarcane aphid (SCA) and reduced aphid landing, respectively (Ikuze et al., 2025). In *Populus deltoides*, the overexpression of *PdCCoAOMT* gene was positively correlated with lignin deposition in transgenic plants compared with the control plants, which highlights the effectiveness of the gene in reducing disease in transgenic tobacco plants (Yadav et al., 2025).

With the rapid development of molecular biology and genomics technology, functional studies of CAD and CCoAOMT gene families have been widely carried out in a variety of plants. In the model plant *Arabidopsis thaliana*, 17 members of the CAD family were found, of which nine genes (*AtCAD1*–*AtCAD9*) had high similarity and consistency with tobacco and loblolly pine (Kim et al., 2004). In rice, there are 12 CAD and CAD-like genes. In order to elucidate the biochemical function of *OsCAD*, *OsCAD1*, *OsCAD2*, *OsCAD6*, and *OsCAD7*, which are highly expressed in rice, were cloned from rice tissues. The expression of *OsCAD2* was induced by *Xanthomonas oryzae* pv. *oryzae* (Xoo), indicating that the gene plays a role in rice defense response (Park et al., 2018). Genome analysis has confirmed that there are 11 and nine CCoAOMT gene members in *Arabidopsis* (Wang et al., 2024) and rice (Yokoyama and Nishitani, 2004), respectively. Although the complete soybean genome sequence was sequenced and published in 2010 (*Williams 82* genome version) (Schmutz et al., 2010), it provides important data support for analyzing the characteristics of gene families at the genome-wide level, but the genome-wide identification of soybean CAD and CCoAOMT gene families has not been reported.

Soybean faces a variety of biotic stresses during the growth process, among which downy mildew caused by *Peronospora manshurica* (*P. manshurica*) is one of the important fungal diseases restricting soybean yield and quality (Roongruangsree et al., 1988). It has been confirmed that the systematic analysis of CAD and CCoAOMT gene families has important application value in the study of plant disease resistance mechanism and provides key targets and technical ideas for crop disease resistance breeding—for example, a total of 21 wheat *TaCCoAOMT* genes were identified in wheat and divided into four groups according to phylogenetic analysis. In addition, 48 RNA-seq samples from *Fusarium graminearum* (*Fg*) infection and four simulated samples of wheat genotypes were used to preliminarily propose potential candidate genes involved in *Fg* response through transcriptome data and qRT-PCR verification (Yang et al., 2021). A total of 54 CAD-like family genes were identified in the three genomes of the *Saccharum* complex. All CAD-like family genes were divided into five phylogenetic groups (I–V). Among them, *SaCAD1*-like1 gene was upregulated in sugarcane varieties after infection with *Xanthomonas albilineans* (*Xa*)-induced leaf burn disease (Wang Z.Q et al., 2025). However, the specific functions and regulatory mechanisms of CAD and CCoAOMT gene families during the process of *P. manshurica* infection in soybean have not been reported.

Based on this, in this study, *GmCAD* and *GmCCoAOMT* gene family members were identified in soybean genome by bioinformatics technology, and their phylogenetic relationship, gene structure, promoter cis-acting elements, and expression patterns under *P. manshurica* infection were analyzed. The purpose of this study is to reveal the molecular regulation mechanism of *GmCAD* and *GmCCoAOMT* gene family in soybean response to downy mildew stress and to provide scientific basis for mining key genes of soybean downy mildew resistance and enriching plant disease resistance molecular breeding theory.

## 2 Materials and methods

### 2.1 Identification and chromosome mapping of *GmCAD* and *GmCCoAOMT* genes in soybean

To systematically identify CAD and CCoAOMT gene family members in soybean, which are key enzymes in the lignin biosynthesis pathway and play critical roles in plant defense against pathogens, we performed genome-wide identification using a homology-based approach. To identify *GmCAD* and *GmCCoAOMT* gene family members in the soybean *Williams 82* genome (Glycine_max_v2.1), protein sequences of seven *Arabidopsis AtCAD* genes and seven *AtCCoAOMT* genes (**Table 1**) were used as query probes. The *GmCAD* and *GmCCoAOMT* genes in the soybean genome *Williams 82* (*Glycine*_*max*_*v2.1*) were searched using the BLASTp program in TBtools-II software (Chen et al., 2023) (*E*-value threshold was set to 1e-10 and the bits value was greater than 50). The soybean sequence is derived from the Phytozome database (https://phytozome-next.jgi.doe.gov/info/Gmax_Wm82_a2_v1), and the *Arabidopsis* sequence is from the Ensembl Plants database (http://plants.ensembl.org/index.html).

**Table 1:** *Arabidopsis AtCAD*/*AtCCoAOMT* query probes for soybean gene family identification.

Gene name	Gene ID	Gene name	Gene ID
*AtCAD1*	*AT1G72680.1*	*AtCCoAOMT1*	*AT4G34050.1*
*AtCAD2*	*AT2G21730.1*	*AtCCoAOMT2*	*AT1G24735.1*
*AtCAD3*	*AT2G21890.1*	*AtCCoAOMT3*	*AT3G61990.1*
*AtCAD4*	*AT3G19450.1*	*AtCCoAOMT4*	*AT3G62000.1*
*AtCAD5*	*AT4G34230.1*	*AtCCoAOMT5*	*AT1G67990.1*
*AtCAD6*	*AT4G37970.1*	*AtCCoAOMT6*	*AT1G67980.1*
*AtCAD7*	*AT4G37980.1*	*AtCCoAOMT7*	*AT4G26220.1*

At the same time, the HMM model files of *GmCAD* family (PF08240, PF00107) and *GmCCoAOMT* family (PF01596) were obtained from the Pfam protein domain database (http://pfam.xfam.org), and HMMER (*E*-value threshold was set to 1e-10 bits value greater than 50) in TBtools-II software was used to identify soybean *GmCAD* and *GmCCoAOMT* gene members. The common protein sequences queried by HMM and Blastp were used to remove redundant protein sequences. To validate the domain integrity of the candidate genes, SMART (https://smart.embl.de/), InterPro (https://www.ebi.ac.uk/interpro/), and NCBI CD-Search (https://www.ncbi.nlm.nih.gov/Structure/bwrpsb/bwrpsb.cgi) websites were used to further verify the integrity of *GmCAD* and *GmCCoAOMT* gene domains. To visualize the genomic distribution of these genes, the Gene Location Visualize from GTF/GFF function in TBtools-II software was used to map the chromosome positions of soybean *GmCAD* and *GmCCoAOMT* gene members.

### 2.2 Physicochemical properties and subcellular localization analysis of *GmCAD* and *GmCCoAOMT* genes in soybean

To characterize the basic biochemical features of the identified *GmCAD* and *GmCCoAOMT* proteins, which may provide insights into their functional properties, the physical and chemical properties of *GmCAD* and *GmCCoAOMT* gene families (including amino acid composition, molecular weight, isoelectric point, instability coefficient, and other physical and chemical properties) were calculated using the Protein Parameter Calc tool in TBtools-II software. To predict where these proteins function within the cell, Wolf PSORT (https://wolfpsort.hgc.jp/) was used to predict the subcellular localization of *GmCAD* and *GmCCoAOMT* gene members.

### 2.3 Phylogenetic tree construction and collinearity analysis of *GmCAD* and *GmCCoAOMT* genes in soybean

To elucidate the evolutionary relationships of *GmCAD* and *GmCCoAOMT* genes across species, a phylogenetic tree was constructed using MEGA11 software based on the protein sequences of soybean *GmCAD* and *GmCCoAOMT* genes and their homologous CAD and CCoAOMT proteins from *Arabidopsis* and rice. The rice sequences were from the Phytozome database (https://phytozome-next.jgi.doe.gov/info/Osativa_v7_0). Firstly, Clustal W was used to perform multiple sequence alignment of protein sequences. The parameters were default. The alignment results were used to construct a phylogenetic tree using the NJ neighbor-joining method, and the bootstrap value (step size test) was set to 1,000 times. The Newick format is exported, and the obtained phylogenetic tree is further processed using Evolview (https://www.evolgenius.info/evolview/#login) (Subramanian et al., 2019).

To investigate gene duplication events and evolutionary selection pressures, the intraspecific collinearity of *GmCAD* and *GmCCoAOMT* genes in soybean was analyzed using MCScanX in TBtools-II software, and the interspecific collinearity among soybean, *Arabidopsis*, and rice was analyzed. Based on the identified collinear gene pairs, the synonymous substitution (Ks), nonsynonymous substitution (Ka) rate, and Ka/Ks ratio were further calculated to evaluate their evolutionary selection pressure. According to the formula T = Ks/2λ (λ = 6.1 × 10^-9^), the separation time (T) of gene pairs with collinearity within species was estimated (Yang et al., 2006).

### 2.4 Analysis of gene structure and conserved motifs of *GmCAD* and *GmCCoAOMT* in soybean

To investigate the structural diversity and evolutionary conservation of *GmCAD* and *GmCCoAOMT* genes, the structural distribution of soybean *GmCAD* and *GmCCoAOMT* gene members was mapped using TBtools-II software. Motif is a short sequence that appears in a group of related genes with certain characteristics, which often contains some transcription factor recognition sites. To identify conserved motifs that may be functionally important, the protein sequence was submitted to MEME (http://meme-suite.org/tools/meme) for motif prediction. The number of motifs was set to eight. The available motifs were screened according to *E*-value less than 0.05. The results were plotted using TBtools-II software.

### 2.5 Promoter prediction of *GmCAD* and *GmCCoAOMT* genes in soybean

To explore the potential transcriptional regulatory mechanisms of *GmCAD* and *GmCCoAOMT* genes, the 2,000-bp sequence upstream of the start codon of *GmCAD* and *GmCCoAOMT* gene members was extracted from the soybean genome *Williams 82* (*Glycine_max_v2.1*) using TBtools-II software as the promoter region. The promoter region sequence was submitted to the PlantCARE (http://bioinformatics.psb.ugent.be/webtools/plantcare/html/) website to analyze the cis-acting elements of the family members, and the TBtools-II software was used for plotting.

### 2.6 miRNA prediction and gene co-expression network analysis of *GmCAD* and *GmCCoAOMT* genes in soybean

To identify potential post-transcriptional regulators of *GmCAD* and *GmCCoAOMT* genes, the CDS sequences of soybean *GmCAD* and *GmCCoAOMT* gene members were extracted using TBtools-II software, and the sequences were uploaded to psRNATarget (https://www.zhaolab.org/psRNATarget) online software to predict the miRNA of soybean family genes. The mathematical expectation is set to 5.0 (Dai et al., 2018).

To understand the functional interactions and collaborative mechanisms among these gene members, the co-expression network of soybean *GmCAD* and *GmCCoAOMT* gene members was constructed using the STRING database (https://cn.string-db.org/cgi/input?sessionId=bjGEFNh5q96x&input_page_show_search=on), and the confidence parameter was set to 0.4 to explore the potential functional association and collaboration mechanism between these gene members.

### 2.7 Expression patterns of *GmCAD* and *GmCCoAOMT* genes in different soybean tissues

To understand the tissue-specific expression profiles of *GmCAD* and *GmCCoAOMT* genes, RNA-seq data from different tissues of *Williams 82* were downloaded from the soybean database (https://www.soybase.org/) (Severin et al., 2010). The FPKM values of *GmCAD* and *GmCCoAOMT* gene members were obtained according to their gene IDs. After homogenization, the expression patterns of *GmCAD* and *GmCCoAOMT* gene members in different tissues were mapped using TBtools-II software.

### 2.8 Expression analysis of soybean *GmCAD* and *GmCCoAOMT* genes under *P. manshurica* stress

To investigate the potential involvement of *GmCAD* and *GmCCoAOMT* genes in soybean defense against *P. manshurica*, expression patterns in response to *P. manshurica* infection were analyzed. In this study, Jiaohe xiaoheidou (JH) with high resistance (HR) to soybean downy mildew and Jilin 5 (JL) with high susceptibility (HS) to soybean downy mildew were selected as experimental materials. The seeds were provided by the Soybean Research Institute of Jilin Academy of Agricultural Sciences and inoculated with *P. manshurica* by artificial inoculation. For JH and JL, soybean leaves were collected at 0 h (CK) and 6, 12, 24, 48, and 72 h after infection with *P. manshurica*, and each treatment was set up with three replicates. The samples were frozen in liquid nitrogen and stored at -80°C.

To validate the RNA-seq results, total RNA was extracted using the EasyPure^®^ Plant RNA Kit (ER301-01). PrimeScript ™ RT reagent Kit with gDNA Eraser (RR047A) was used to synthesize cDNA. The specific qRT-PCR primers (**Supplementary Table 1**) were designed using https://www.primer3plus.com/. The primer length was 18–22 bp, the annealing temperature was 55–60°C, and the amplified fragment length was 100–200 bp. The candidate *GmCAD* and *GmCCoAOMT* gene members were amplified by real-time RT-PCR on the CFX48 ECO TM Real-time PCR system (Illumina). The reaction system comprised 5 μL TB Green premix, 0.2 μM upstream and downstream primers, 60 ng cDNA, and ultrapure water supplemented at 10 μL. The reaction conditions were 50°C for 2 min, 95°C for 5 min, 95°C for 10 s, 55°C–60°C for 30 s, and 72°C for 1 min for a total of 40 cycles. All experiments maintained three independent biological replicates. The relative expression of the target gene was calculated using the 2^-ΔΔCT^ method, and the difference in the expression level of the candidate gene was analyzed using GraphPad Prism 8 software. In addition, the FPKM value of transcriptome data (sequencing data completed in the early stage of this study) and the relative expression obtained by qRT-PCR were plotted using TBtools-II software.

### 2.9 Determination of lignin content and analysis of lignin pathway expression patterns

To assess the functional relevance of *GmCAD* and *GmCCoAOMT* genes in lignin-mediated defense, lignin content was measured and the expression patterns of the entire lignin biosynthesis pathway were examined. After infection with *P. manshurica*, JH and JL were used as materials. Soybean leaves were collected from uninfected (control, CK) and *P. manshurica*-infected plants at 6, 12, 24, 48, and 72 h post-inoculation, with three biological replicates per treatment. The lignin content of the samples was determined using the Lignin Content Extraction Kit (M1711B) from Suzhou Monxi Biomedical Technology Co., Ltd., to compare the differences in lignin content between JH and JL before and after *P. manshurica* infection. Meanwhile, the overall expression regulatory characteristics of the lignin biosynthesis pathway during soybean’s response to *P. manshurica* infection were investigated. Based on the previous transcriptome sequencing data, this study normalized the expression levels of key enzyme genes involved in the core lignin biosynthesis pathway (including PAL, C4H, 4CL, HCT, C3H, F5H, COMT, and CCR) using log2(FPKM + 1), and heatmaps of their expression pattern were constructed using TBtools-II software.

## 3 Results

### 3.1 Identification and chromosome mapping of *GmCAD* and *GmCCoAOMT* genes in soybean

The protein sequences of seven *Arabidopsis AtCAD* genes and seven *Arabidopsis AtCCoAOMT* genes were used as probes, and the *GmCAD* and *GmCCoAOMT* genes were identified using TBtools-II software BLASTp and HMMER tools. After removing the redundancy of the common results of the two methods, the domain integrity was verified using SMART, InterPro, and NCBI CD-Search websites. Finally, 41 soybean *GmCAD* gene members (**Supplementary Table 2**) and 13 *GmCCoAOMT* gene members (**Supplementary Table 3**) were obtained. TBtools-II software was used to analyze the chromosome distribution of *GmCAD* and *GmCCoAOMT* gene members in soybean. It was found that 41 *GmCAD* gene members were distributed on 19 chromosomes of soybean, including Chr1, Chr2, and Chr3 (**Supplementary Figure 1**), and 13 *GmCCoAOMT* gene members were distributed on seven chromosomes, including Chr1, Chr2, Chr5, Chr7, Chr8, Chr11, and Chr17 (**Supplementary Figure 2**).

### 3.2 Physicochemical properties and subcellular localization analysis of *GmCAD* and *GmCCoAOMT* genes in soybean

The physicochemical properties of soybean *GmCAD* and *GmCCoAOMT* genes were predicted using TBtools-II. The results showed that the amino acid number of 41 *GmCAD* family members was 305–461 aa, the molecular weight was 32,980.96–50,658.88 u, and the theoretical isoelectric point was 5.51–8.78. There were four proteins with isoelectric point greater than seven, indicating that 9.76% of *GmCAD* proteins were alkaline proteins, and the instability coefficient was between 18.74 and 42.73. There are five *GmCAD* proteins with instability coefficient higher than 40, which are unstable proteins. The fat coefficient of the protein is between 79.76 and 99.82, and the total average hydrophilicity value is positive, indicating that the *GmCAD* protein belongs to hydrophobic proteins (**Supplementary Table 2**).

The results of the subcellular localization showed that 23 *GmCAD* genes were predicted to be located in the cytoplasm (cyto), seven in the chloroplast (chlo), four in the cytoskeleton (cysk), two in the vacuole (vacu), one in the endoplasmic reticulum (ER), and one in the extracellular space (extr). Moreover, *GmCAD38* may be located in the cyto and chlo, *GmCAD36* may be located in the chlo and peroxisome (pero), and *GmCAD11* may be located in the chlo and chlo-mitochondrion (mito) (**Supplementary Table 2**).

The number of amino acids of the 13 *GmCCoAOMT* gene members was 193–325 aa, the molecular weight was 21,623.83–36,972.2 u, and the theoretical isoelectric point was 5.01–8.67. One protein with isoelectric point greater than seven was an alkaline protein, and the instability coefficient was between 33.21 and 51.29. There were seven *GmCCoAOMT* proteins with instability coefficient higher than 40, which were unstable proteins. The fat coefficient of the protein was between 86.74 and 107.64, and the total average hydrophilicity value was negative. The results showed that *GmCCoAOMT* protein was a hydrophilic protein (**Supplementary Table 3**). The subcellular localization results showed that 10 *GmCCoAOMT* genes were predicted to be located in the cyto, two in the chloro, and one in the cyto-nucleus (nucl) (**Supplementary Table 3**).

### 3.3 Phylogenetic tree construction and collinearity analysis of *GmCAD* and *GmCCoAOMT* genes in soybean

**Figure 1:**
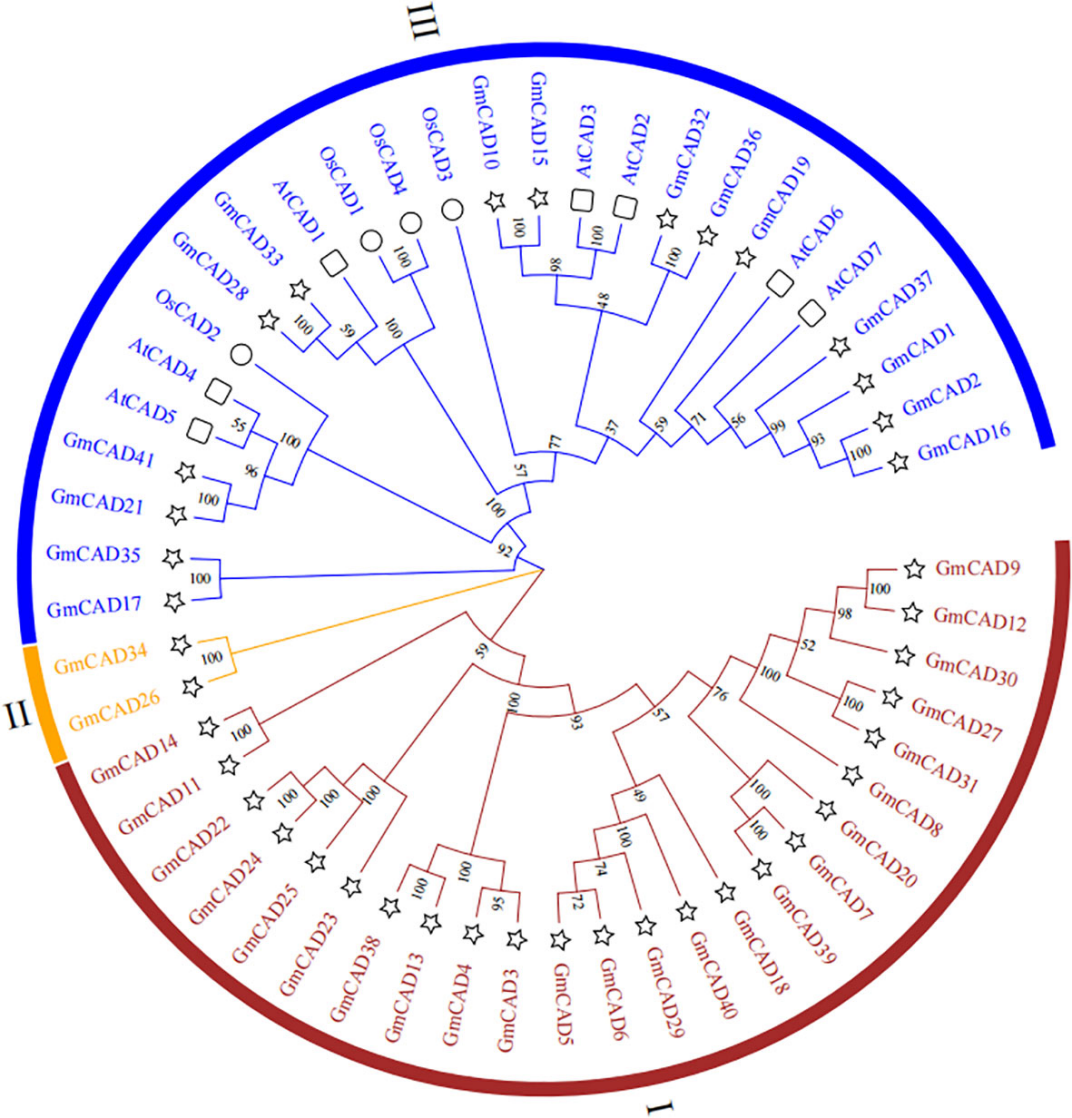
Phylogenetic tree analysis of soybean *GmCAD* gene with *Arabidopsis* and rice. This phylogenetic tree was constructed using the neighbor-joining method with 1,000 bootstrap replicates. Based on the evolutionary relationships, the *GmCAD* proteins were divided into three distinct clusters (I–III).

Using the MEGA11 software, 52 CAD protein sequences were identified, including 41 *GmCAD* from soybean, seven *AtCAD* from *Arabidopsis thaliana*—including *AT1G72680.1* (*AtCAD1*), *AT2G21730.1* (*AtCAD2*), *AT2G21890.1* (*AtCAD3*), *AT3G19450.1* (*AtCAD4*), *AT4G34230.1* (*AtCAD5*), *AT4G37970.1* (*AtCAD6*), and *AT4G37980.1* (*AtCAD7*)—and four *OsCAD* genes in rice including *LOC_Os10g11810* (*OsCAD1*), *LOC_Os02g09490* (*OsCAD2*), *LOC_Os10g29470* (*OsCAD3*), and *LOC_Os11g40690* (*OsCAD4*), of which the soybean sequences derived from the Phytozome database (https://phytozome-next.jgi.doe.gov/info/Gmax_Wm82_a2_v1), the *Arabidopsis* sequences from the Ensembl Plants database (http://plants.ensembl.org/index.html), and the rice sequences from the Phytozome database (https://phytozome-next.jgi.doe.gov/info/Osativa_v7_0) were subjected to multiple alignments, and a phylogenetic tree was constructed (**Figure 1**).

The results showed that the CAD gene members were divided into three subgroups. There were 24 members in subgroup I, all of which were soybean *GmCAD* genes, two members in subgroup II, *GmCAD26* and *GmCAD34*, and 26 members in subgroup III, including soybean, *Arabidopsis*, and rice.

**Figure 2:**
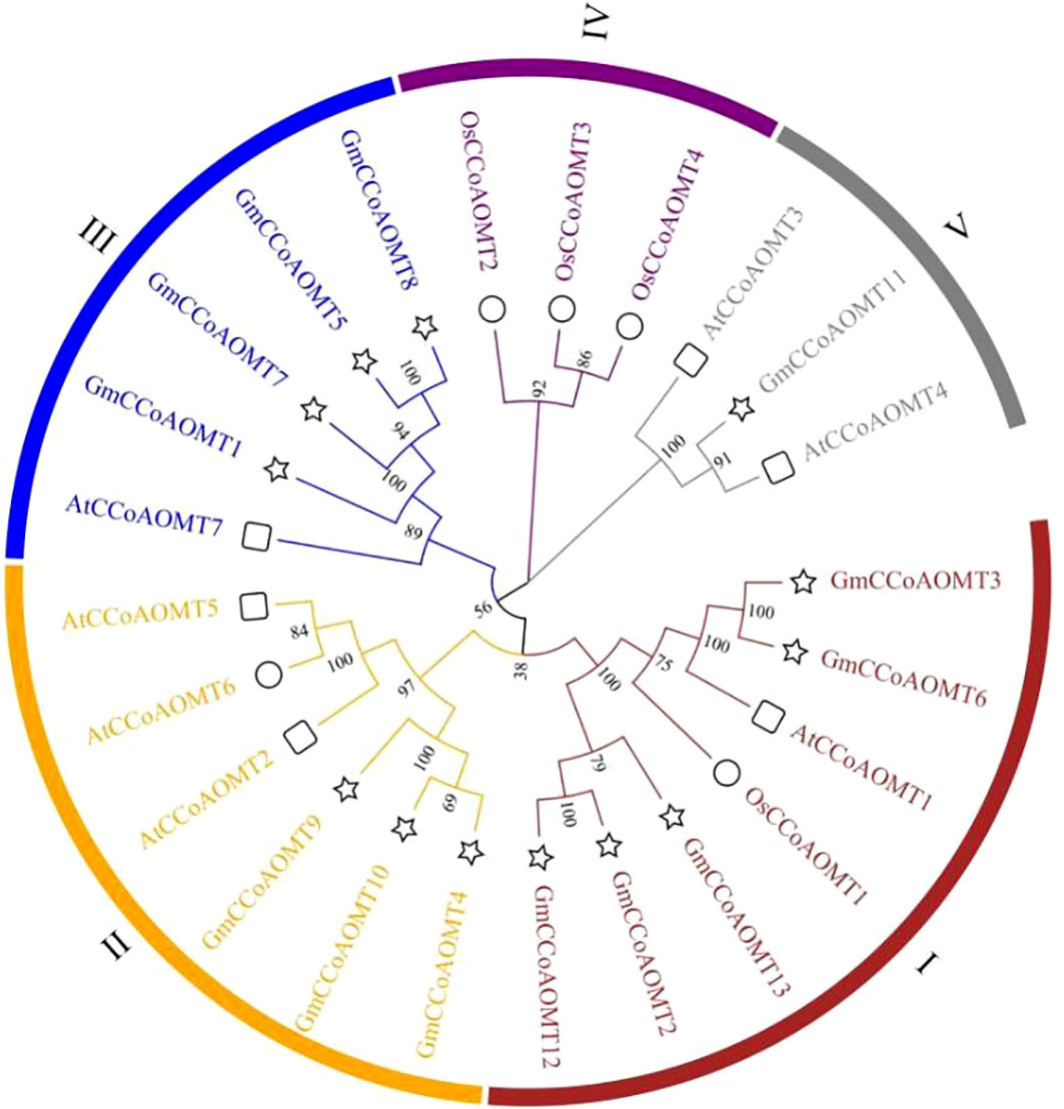
Phylogenetic tree analysis of soybean *GmCCoAOMT* gene with *Arabidopsis* and rice. This phylogenetic tree was constructed using the neighbor-joining method with 1,000 bootstrap replicates. Based on the evolutionary relationships, the *GmCCoAOMT* proteins were divided into five distinct clusters (I–V).

MEGA11 was used to analyze 13 *GmCCoAOMT* genes in soybean and seven *AtCCoAOMT* genes in *Arabidopsis*. It includes *AT4G34050.1* (*AtCCoAOMT1*), *AT1G24735.1* (*AtCCoAOMT2*), *AT3G61990.1* (*AtCCoAOMT3*), *AT3G62000.1* (*AtCCoAOMT4*), *AT1G67990.1* (*AtCCoAOMT5*), *AT1G67980.1* (*AtCCoAOMT6*), and *AT4G26220.1* (*AtCCoAOMT7*) as well as four *OsCCoAOMT* genes in rice including *LOC_Os06g06980* (*OsCCoAOMT1*), *LOC_Os09g30360* (*OsCCoAOMT2*), *LOC_Os08g38910* (*OsCCoAOMT3*), and *LOC_Os08g38920* (*OsCCoAOMT4*). A total of 24 CCoAOMT protein sequences were used to construct a phylogenetic tree (**Figure 2**). The results showed that the CCoAOMT gene members were divided into five subgroups, with seven members in subgroup I, six members in subgroup II, five members in subgroup III, three members in subgroup IV, and three members in subgroup V. Subgroups I, II, III, and V all contain *Arabidopsis* genes, and subgroup IV consist entirely of rice genes, indicating that the soybean *GmCCoAOMT* gene has a stronger evolutionary correlation with the dicotyledonous plant *Arabidopsis*, sharing the conserved functional modules unique to dicotyledonous plants.

**Figure 3:**
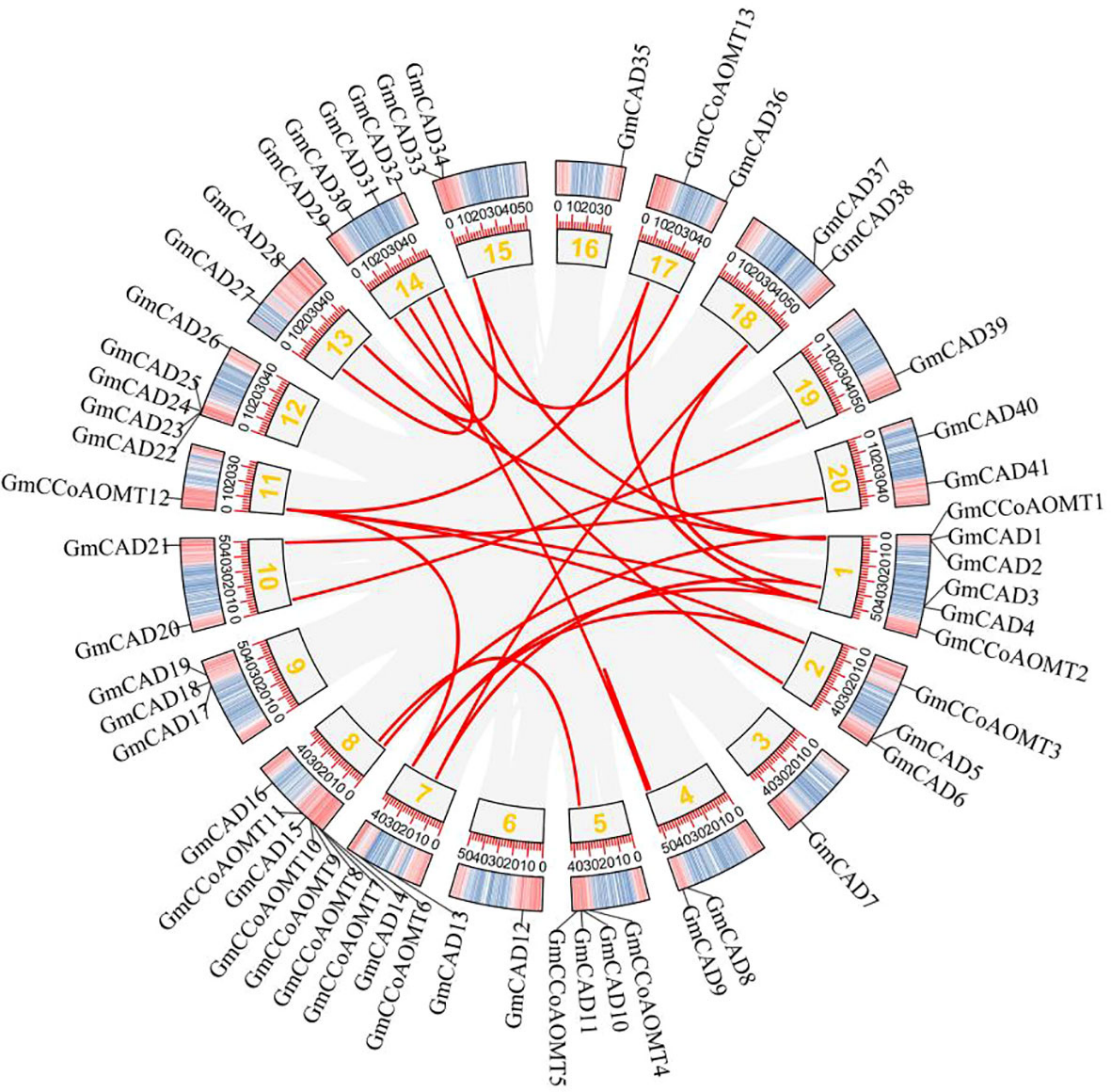
Intraspecific collinearity analysis of *GmCAD* and *GmCCoAOMT* genes in soybean. The red lines indicate the gene pairs that are duplicated in the fragments, while the gray lines represent the homologous blocks in the soybean genome; the outer ring and the heatmap show the distribution of gene density on each chromosome.

In order to clarify the evolutionary relationship between *GmCAD* and *GmCCoAOMT* genes in soybean, a collinearity analysis of *GmCAD* and *GmCCoAOMT* genes was carried out (**Figure 3**). It was found that there were 13 pairs of homologous genes in the *GmCAD* gene, of which 12 pairs of homologous genes have Ka/Ks values (the ratio of non-synonymous substitution number of non-synonymous substitution sites to synonymous substitution number of synonymous substitution sites) less than 1, which was a purification selection. The gene function was highly conserved, and only one pair of *GmCAD1* and *GmCAD28* had no Ka/Ks value. The separation time of the *GmCAD* gene pair was between 6.25 (*GmCAD21* and *GmCAD41*) and 251.88 (*GmCAD1* and *GmCAD33*) Mya (million years ago) (**Supplementary Table 4**). The GmCCoAOMT gene has nine pairs of homologous genes, and the Ka/Ks value is less than 1, all of which are under purifying selection. The separation time of *GmCCoAOMT* gene pairs ranged from 9.95 (*GmCCoAOMT2* and *GmCCoAOMT12*) to 128.54 (*GmCCoAOMT12* and *GmCCoAOMT3*) Mya (million years ago) (**Supplementary Table 4**).

**Figure 4:**
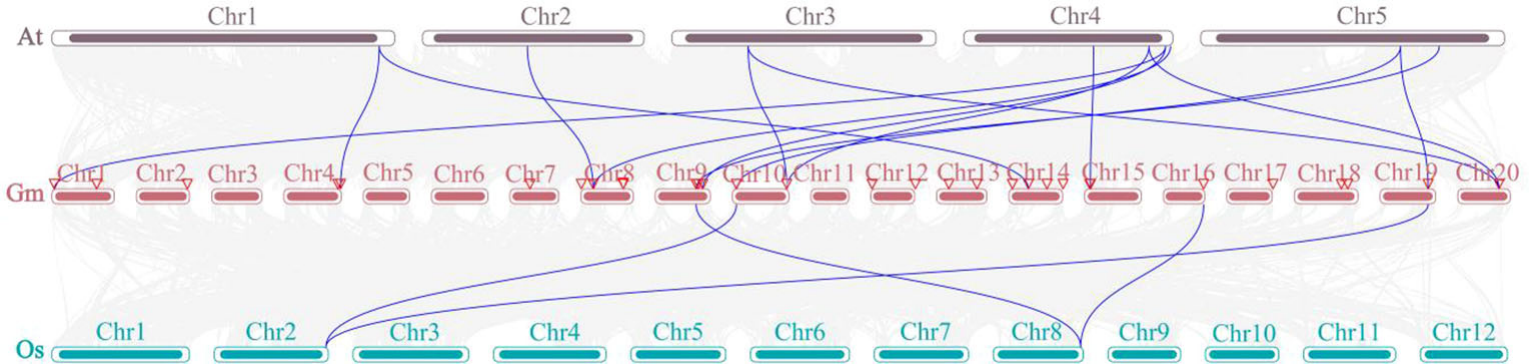
Interspecific collinearity analysis of *GmCAD* genes in soybean, *Arabidopsis*, and rice. The blue lines represent the homologous gene pairs between soybean and *Arabidopsis thaliana* as well as rice, while the gray lines indicate the homologous regions between the soybean genome and those of *Arabidopsis thaliana* and rice.

**Figure 5:**
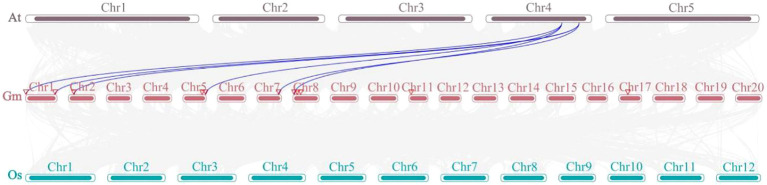
Interspecific collinearity analysis of *GmCCoAOMT* genes in soybean, *Arabidopsis*, and rice. The blue lines represent the homologous gene pairs between soybean and *Arabidopsis thaliana*, while the gray lines indicate the homologous regions between the genomes of soybean, *Arabidopsis thaliana*, and rice.

In the analysis of collinearity between soybean and other species, there are 14 pairs of homologous genes between soybean *GmCAD* and *Arabidopsis AtCAD* genes and four pairs of CAD homologous genes between soybean *GmCAD* and rice *OsCAD* genes (**Figure 4**). It can be seen that the collinearity between soybean *GmCAD* and *Arabidopsis AtCAD* genes is higher. There are six pairs of homologous genes between soybean *GmCCoAOMT* and *Arabidopsis AtCAD* genes (**Figure 5**), and there is no homologous gene between soybean *GmCCoAOM*T and rice *OsCAD* genes, indicating that the gene family may undergo lineage-specific evolution or functional differentiation after monocotyledonous and dicotyledonous plant differentiation.

### 3.4 Analysis of gene structure and conserved motifs of *GmCAD* and *GmCCoAOMT* in soybean

**Figure 6:**
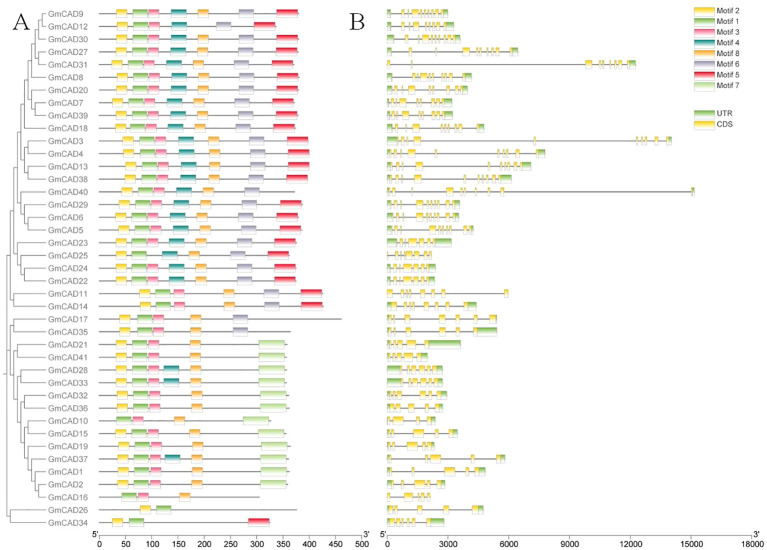
Analysis of gene structures and conserved motifs of *GmCAD* genes in soybean. **(A)** Conserved motifs of *GmCAD* genes. The colored squares represent different motifs. **(B)** Gene structure analysis showing the exon–intron arrangement. The colored squares represent different UTRs and CDSs.

In order to clarify the evolutionary relationship between *GmCAD* and *GmCCoAOMT* genes in soybean, an evolutionary tree was constructed using full-length proteins, and the structure and conserved motifs of *GmCAD* and *GmCCoAOMT* genes in soybean were analyzed using TBtools-II. The 41 *GmCAD* gene members were divided into three subgroups. The analysis of intron and exon information of *GmCAD* gene members showed that the number of exons was five to 10, and it could be seen that the genes with similar positions in the evolutionary tree had similar structures (**Figure 6**). The results of the MEME analysis of conserved motifs of CAD family members showed that a total of eight conserved motifs of *GmCAD* protein were obtained (**Figure 6**) and, in turn, named as motif 1–motif 8. Each subfamily has its own unique motifs and motif combinations, and the same subfamily has the same or similar conserved motifs.

**Figure 7:**
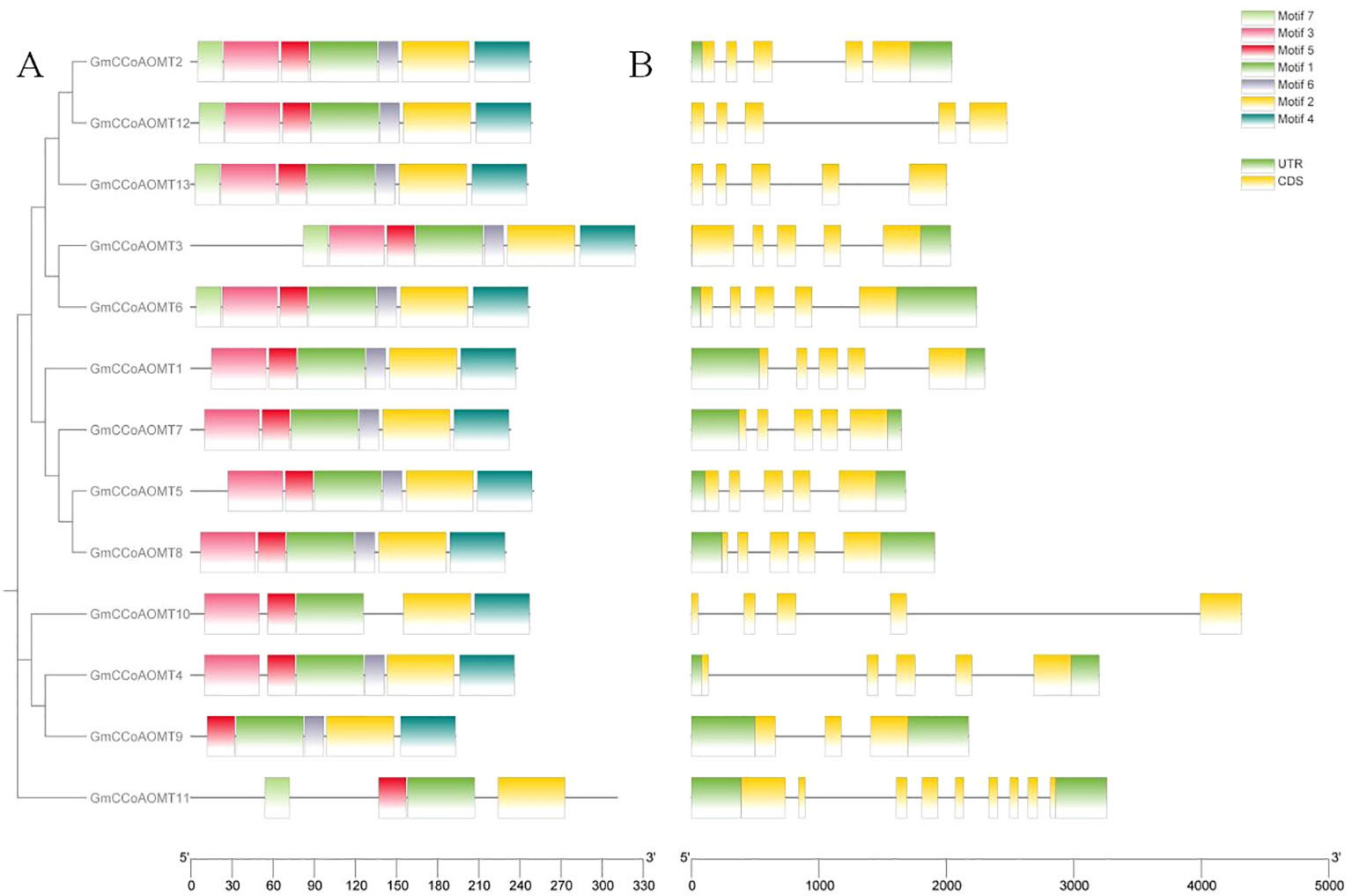
Analysis of gene structures and conserved motifs of *GmCCoAOMT* genes in soybean. **(A)** Conserved motifs of *GmCCoAOMT* genes. The colored squares represent different motifs. **(B)** Gene structure analysis showing the exon–intron arrangement. The colored squares represent different UTRs and CDSs.

The 13 *GmCCoAOMT* gene members were divided into four subgroups. The analysis of intron and exon information of *GmCCoAOMT* gene members showed that the number of exons was three to nine. It can be seen that the genes with similar positions in the phylogenetic tree have similar structures (**Figure 7**). The results of the MEME analysis of conserved motifs of *GmCCoAOMT* family members showed that a total of seven conserved motifs of *GmCCoAOMT* protein were obtained (**Figure 7**), subsequently named motif 1–motif 7. Each subfamily has its own unique motifs and motif combinations, and the same subfamily has the same or similar conserved motifs.

### 3.5 Promoter prediction of *GmCAD* and *GmCCoAOMT* genes in soybean

**Figure 8:**
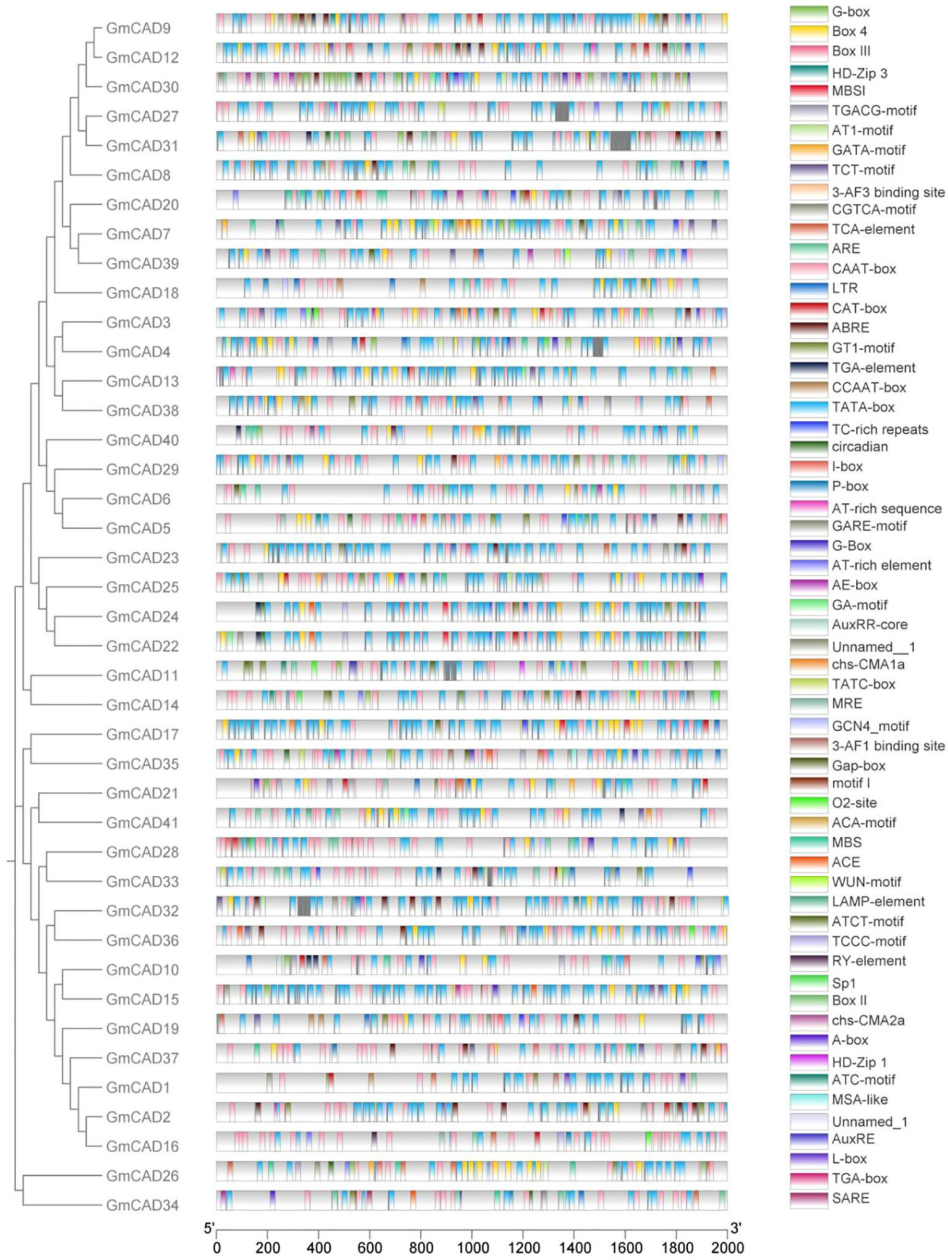
Analysis of cis-acting elements of *GmCAD* gene promoter in soybean. The colored squares on the right represent various types of cis-regulatory elements involved in different biological processes.

The cis-acting elements of soybean *GmCAD* and *GmCCoAOMT* gene promoters were predicted using the online tool PlantCARE. The 41 *GmCAD* gene members contained different numbers and types of cis-acting elements (**Figure 8**). Each *GmCAD* promoter contained CAAT-box and TATA-box elements. A total of 56 types of core promoter elements were identified and divided into six categories according to functions (**Supplementary Table 5**), including abiotic stress responsive (I), core (II), light responsive (III), phytohormone responsive (IV), plant growth (V), and TF recognition and binding site (VI).

**Figure 9:**
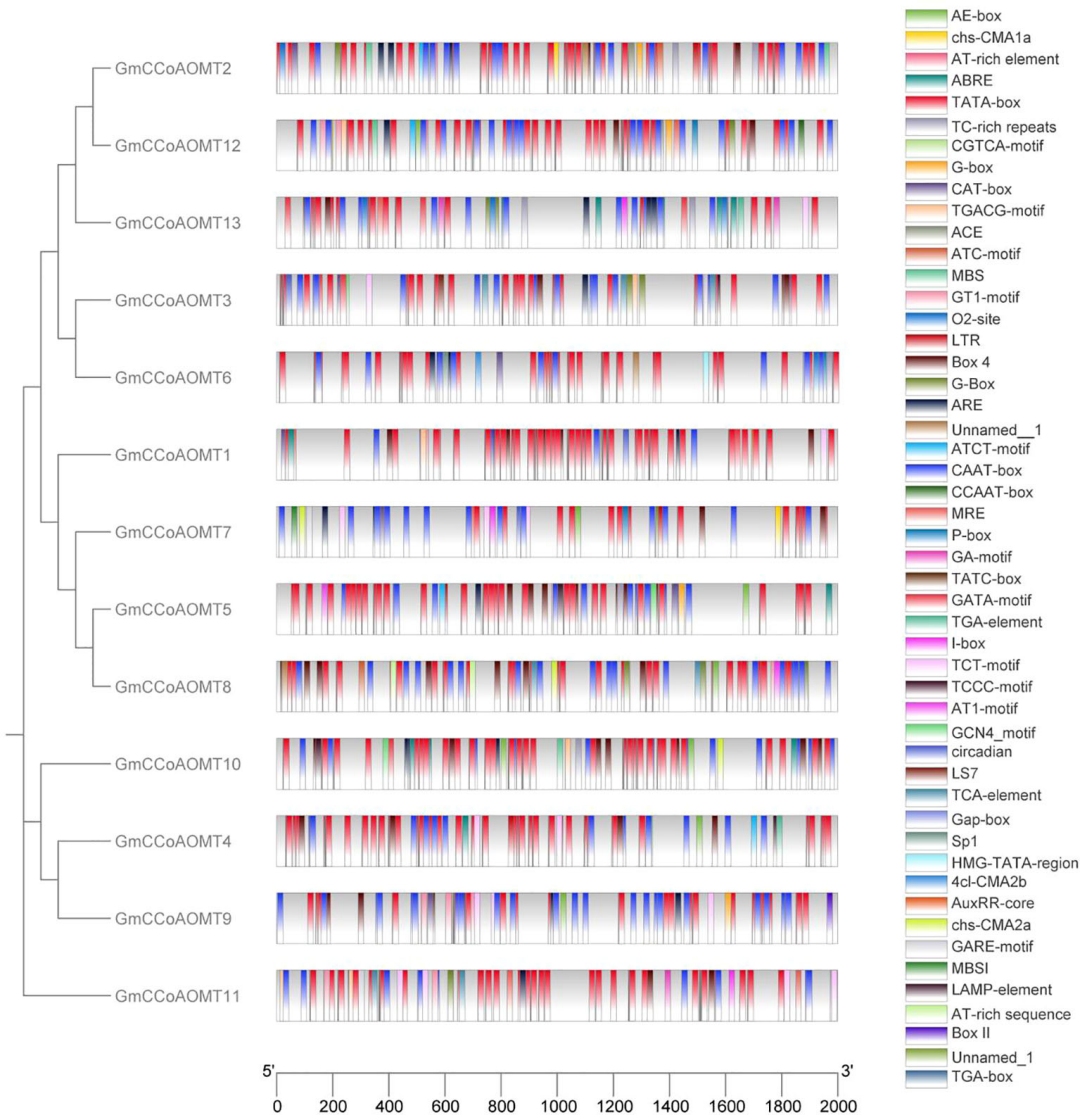
Analysis of cis-acting elements of *GmCCoAOMT* gene promoter in soybean. The colored squares on the right represent various types of cis-regulatory elements involved in different biological processes.

The 13 *GmCCoAOMT* family members all contain a large number of cis-acting elements of different numbers and types (**Figure 9**). Each *GmCCoAOMT* promoter contains CAAT-box and TATA-box elements. A total of 46 types of core promoter elements were identified and divided into six categories according to their functions (**Supplementary Table 6**), including abiotic stress responsive (I), core (II), light responsive (III), phytohormone responsive (IV), plant growth (V), and TF recognition and binding site (VI).

### 3.6 miRNA prediction and gene co-expression network analysis of *GmCAD* and *GmCCoAOMT* genes in soybean

miRNA is involved in the regulation of post-transcriptional gene expression and is an important regulatory element in plants. The psRNATarget online software was used to predict the miRNAs that regulate the coding regions of *GmCAD* and *GmCCoAOMT* genes. As shown in **Supplementary Table 7**, 161 miRNAs were identified to interact with 13 *GmCAD* gene members. *GmCAD6* interacts with 26 miRNAs, while the *GmCAD28* and *GmCAD37* genes interact with only one miRNA. It was also found that one or more miRNAs regulated one or more target genes, and 92 miRNAs regulated one *GmCAD*, such as gma-miR1507c-5p, gma-miR1509a, and gma-miR1509b. Three miRNAs regulated eight *GmCAD*, such as gma-miR172h-5p, gma-miR172i-5p, and gma-miR172j. A small number of miRNAs can simultaneously regulate different *GmCAD* genes, and most miRNAs only regulate one *GmCAD* target gene.

In the *GmCCoAOMT* family, 39 miRNAs were identified to interact with 13 *GmCCoAOMT* gene members (**Supplementary Table 8**). *GmCCoAOMT4* interacts with 11 miRNAs, while the *GmCCoAOMT1*, *GmCCoAOMT5*, and *GmCCoAOMT8* genes interact with only one miRNA. It was also found that one or more miRNAs regulated one or more target genes, and 34 miRNAs regulated one *GmCCoAOMT*, such as gma-miR1516c, gma-miR1534, and gma-miR156k. Four miRNAs regulated two *GmCCoAOMT*, such as gma-miR4993, gma-miR5032, gma-miR9744, gma-miR9749, and gma-miR4379, which regulated three target genes. A small number of miRNAs can simultaneously regulate different *GmCCoAOMT* genes, and most miRNAs only regulate one *GmCCoAOMT* target gene.

**Figure 10:**
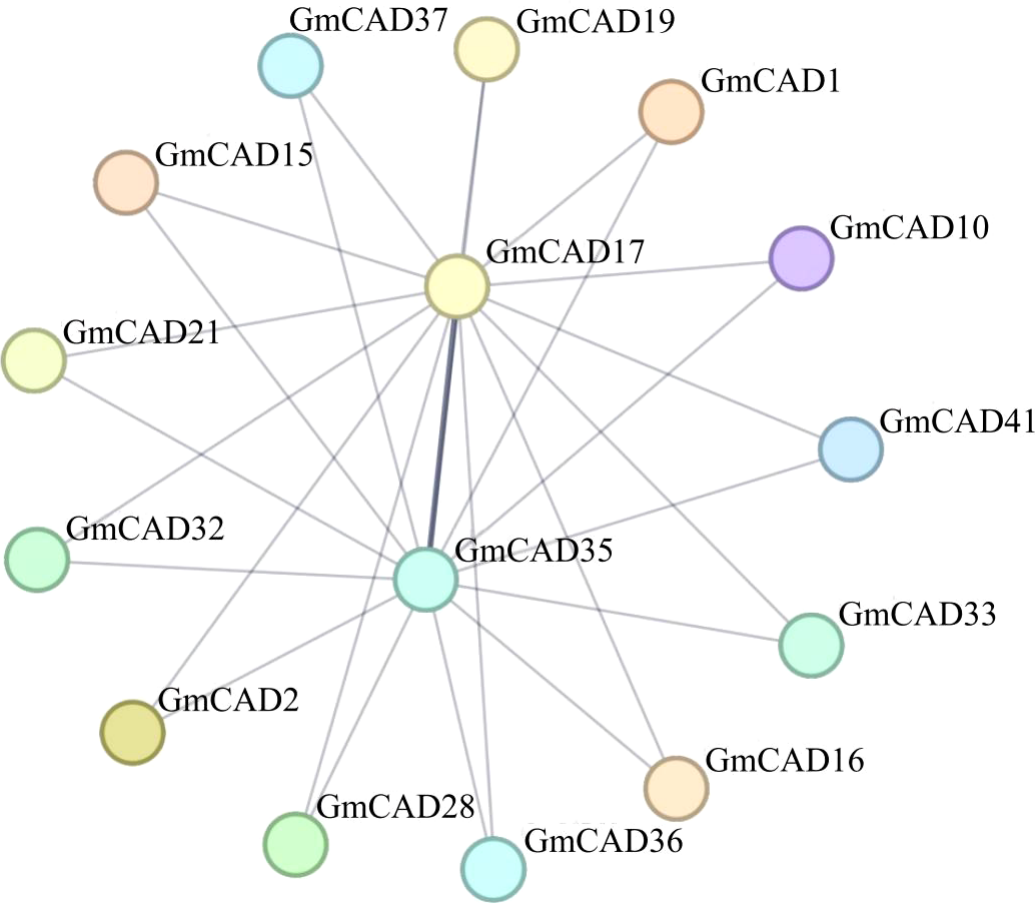
Protein–protein interaction regulatory network of soybean *GmCAD* gene members. Each node represents a *GmCAD* protein, and the multi-colored edges represent the interactions between the proteins.

The co-expression network of soybean *GmCAD* and *GmCCoAOMT* gene members was constructed using the STRING database (**Figure 10**). It was found that there were interactions among members of the soybean *GmCAD* gene family. *GmCAD17* and *GmCAD35* interacted with 13 *GmCAD* gene members, including *GmCAD1*, *GmCAD2*, *GmCAD10*, *GmCAD15*, *GmCAD16*, *GmCAD19*, *GmCAD21*, *GmCAD28*, *GmCAD32*, *GmCAD33*, *GmCAD36*, *GmCAD37*, and *GmCAD41*. There was no interaction within the soybean CCoAOMT gene members, and there was no interaction between *GmCAD* and *GmCCoAOMT* gene members. This indicates that there is a wide range of interactions between soybean *GmCAD* gene members, and some members, *GmCAD17* and *GmCAD35*, may be the core members of the family, while *GmCCoAOMT* gene members have no internal interaction and no interaction between the two families, reflecting that there are significant differences in the interaction patterns between the two gene families.

### 3.7 Expression pattern analysis of *GmCAD* and *GmCCoAOMT* genes in different tissues of soybean

The RNA-seq data of different tissues of *Williams 82* were downloaded from the soybean database (https://www.soybase.org/). The fragments per kilobase of exon model per million mapped fragments (FPKM) values were extracted according to the gene IDs of *GmCAD* and *GmCCoAOMT* gene members. After standardization with log2(FPKM + 1), the expression patterns of the two gene family members in different tissues were plotted using TBtools-II software.

**Figure 11:**
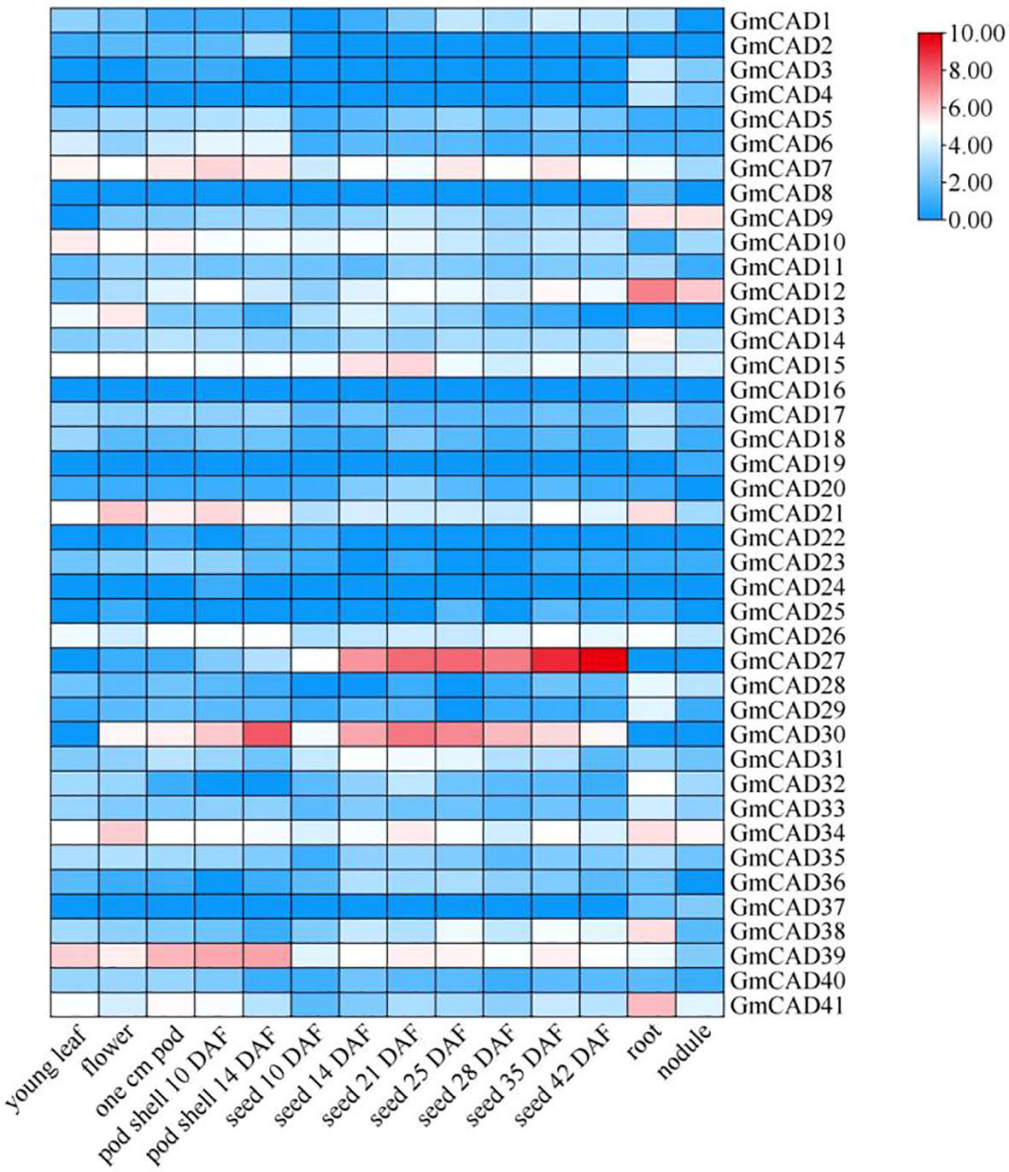
Expression pattern analysis of *GmCAD* genes in different tissues of soybean. The expression values are plotted using log2(FPKM + 1). From blue (low expression) to red (high expression).

**Figure 12:**
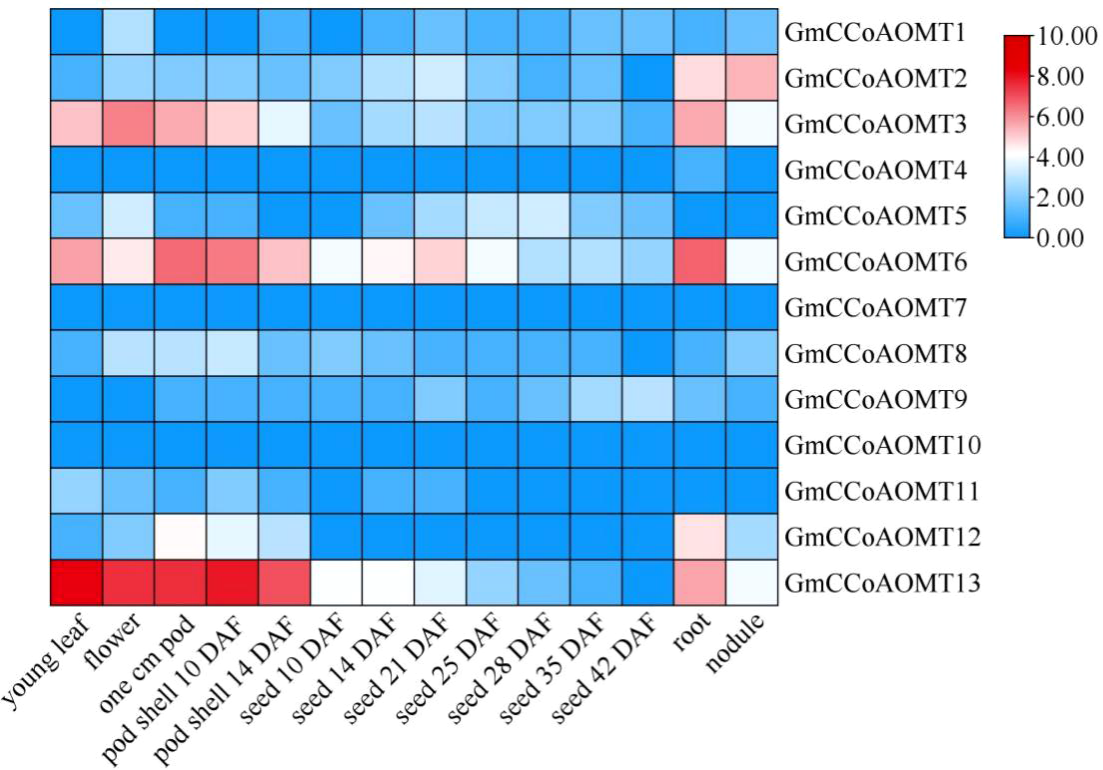
Expression pattern analysis of *GmCCoAOMT* genes in different tissues of soybean. The expression values are plotted using log2(FPKM + 1). From blue (low expression) to red (high expression).

The expression patterns of *GmCAD* gene members showed significant differences (**Figure 11**). *GmCAD16* was not expressed in all tissues, while *GmCAD27* is highly expressed in seed 35 DAF and seed 42 DAF and is lowly expressed in tissues such as roots and nodules. *GmCAD30* is highly expressed in pod shell 14 DAF and is lowly expressed in tissues such as young leaves, roots, and nodules. The expression of this family shows strong specificity for the developmental stages of the organism. *GmCCoAOMT7* and *GmCCoAOMT10* genes were not expressed in all tissues (**Figure 12**), while GmCCoAOMT13 is highly expressed in young leaves, flowers, and 1-cm pods. It is expressed at a low level in seeds at 42 DAF, indicating that the expression of *GmCCoAOMT* gene members also showed obvious tissue specificity.

### 3.8 Expression patterns of *GmCAD* and *GmCCoAOMT* genes and lignin accumulation in soybean during *P. manshurica* infection

**Figure 13:**
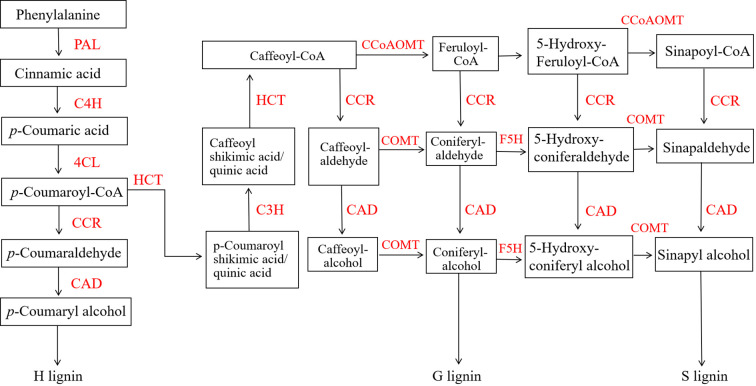
Schematic diagram of the lignin biosynthesis pathway in soybean. The pathway depicts the metabolic route from phenylalanine to the three primary monolignols (*p*-coumaryl alcohol, coniferyl alcohol, and sinapyl alcohol), which polymerize into H-lignin, G-lignin, and S-lignin. Enzymes catalyzing each reaction step are indicated in red text.

**Figure 14:**
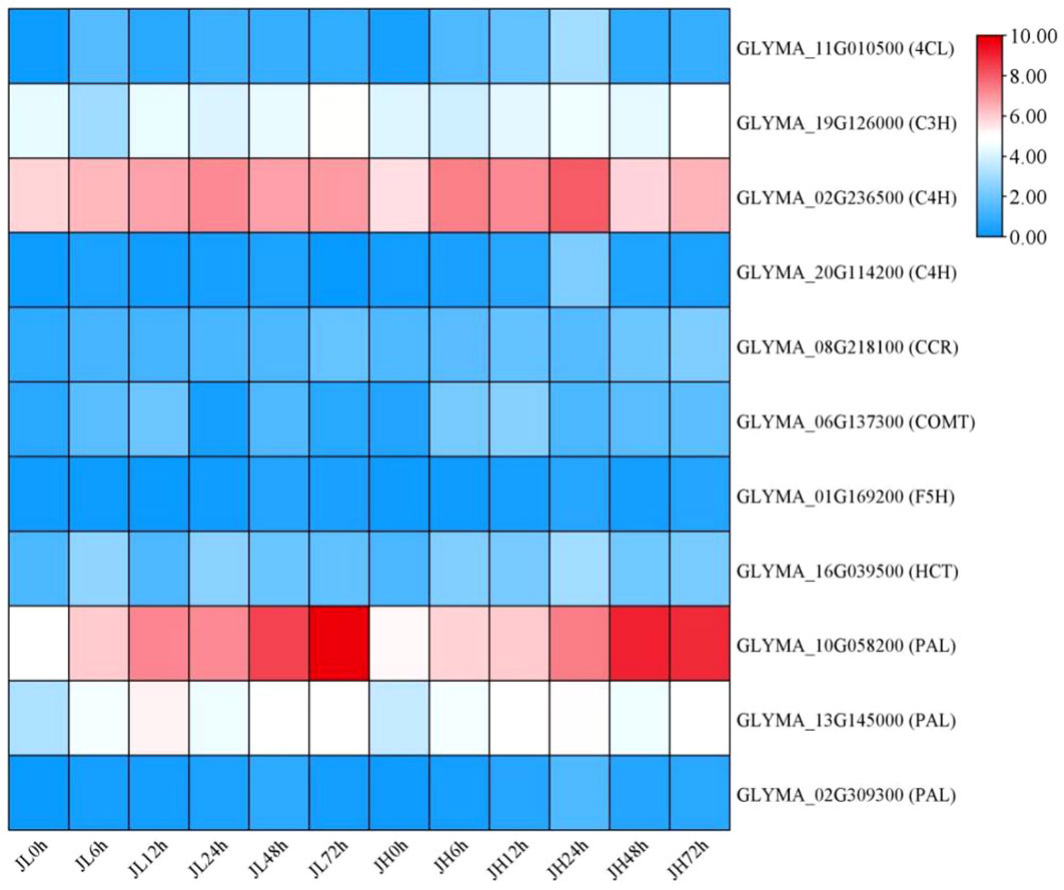
Analysis of the expression patterns of genes related to the lignin synthesis pathway under *P. manshurica* infection. The expression values are plotted using log2(FPKM + 1). From blue (low expression) to red (high expression).

To reveal the overall expression regulatory characteristics of the lignin biosynthesis pathway during soybean’s response to *P. manshurica* infection, this study, based on the previous transcriptome sequencing data, constructed a heatmap of the expression patterns of the core pathway of lignin monomer synthesis and key genes (**Figure 13**). Lignin biosynthesis starts with phenylalanine as the initial substrate, which is catalyzed by upstream key enzymes PAL, C4H, and 4CL to generate *p*-coumaroyl-CoA; then, it is successively catalyzed by HCT, C3H, CCoAOMT, F5H, and COMT to form three monomers—namely: *p*-coumaryl alcohol, coniferyl-alcohol, and sinapyl alcohol—and finally polymerized to form H, G, and S types of lignin. The expression heatmap results (**Figure 14**) show that the expression characteristics of each key enzyme gene in the pathway in response to *P. manshurica* infection vary significantly. Among them, C4H and some PAL significantly upregulated in the later stage of infection, which is consistent with the overall activation trend of the lignin synthesis pathway, while the expression levels of F5H, CCR, COMT, etc., were overall low and changed gradually and were not the main regulatory nodes under the conditions of this study. The combination of the lignin pathway diagram and the gene expression heatmap clearly presents the catalytic sites and transcriptional response patterns of each key enzyme, providing an important basis for screening candidate genes involved in the defense response against *P. manshurica* infection.

To further explore whether the key family genes *GmCAD* and *GmCCoAOMT* of lignin synthesis are involved in the defense process of soybean against *P. manshurica* infection, qRT-PCR was used to detect the expression levels of *GmCAD* and *GmCCoAOMT* family genes at different infection time points in the resistant material JH and the susceptible material JL, and the visualization drawing was completed using TBtools-II software.

**Figure 15:**
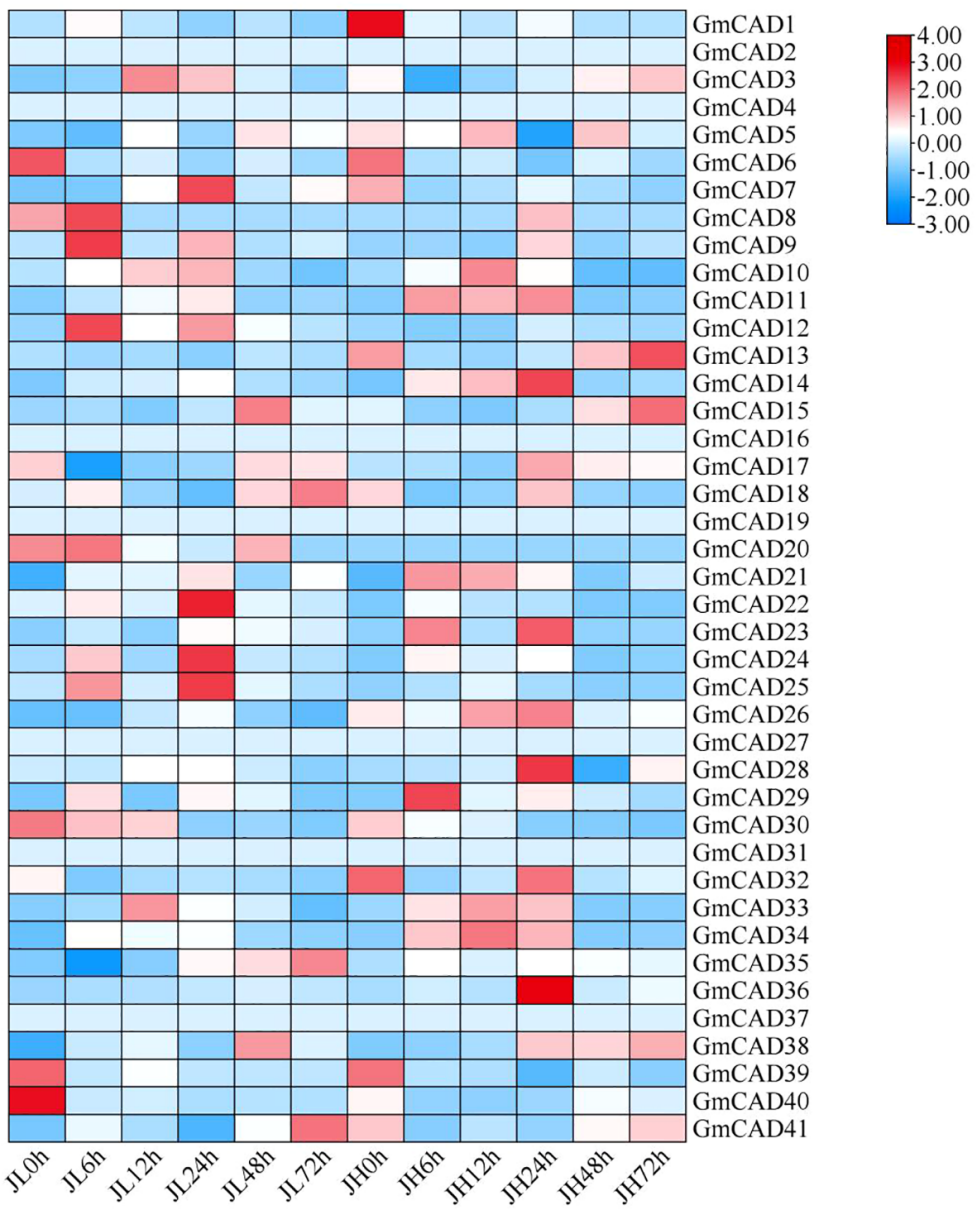
Expression pattern analysis of *GmCAD* gene in soybean under *P. manshurica* infection. The expression values in this figure represent relative gene expression levels obtained from qRT-PCR analysis. From blue (low expression) to red (high expression).

The expression results of the *GmCAD* family in soybeans are shown in **Figure 15**: *GmCAD2*, *GmCAD4*, *GmCAD16*, *GmCAD19*, *GmCAD27*, and *GmCAD37* showed almost no expression in the tested materials. *GmCAD14*, *GmCAD26*, *GmCAD28*, and *GmCAD36* exhibited an increasing and then decreasing expression trend at 6, 12, 24, 48, and 72 h after the infection of the disease-resistant material JH, reaching the peak expression at 24 h, while there was no significant expression fluctuation in the susceptible material JL. *GmCAD29* showed a significantly higher expression level at 6 h after the infection of JH compared to other time points, and there was no significant change in JL.

**Figure 16:**
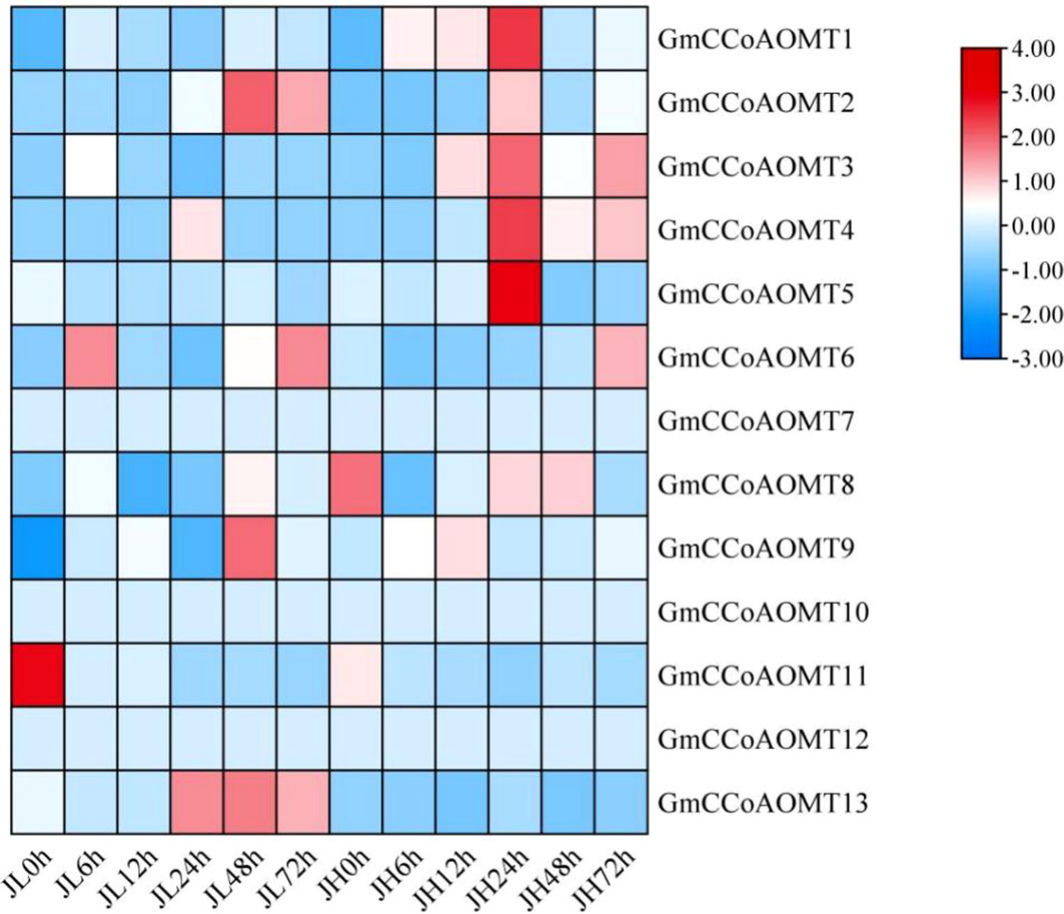
Expression pattern of *GmCCoAOMT* genes in soybean under *P. manshurica* infection. The expression values in this figure represent relative gene expression levels obtained from qRT-PCR analysis. From blue (low expression) to red (high expression).

The expression results of the *GmCCoAOMT* family in soybeans are shown in **Figure 16**: *GmCCoAOMT7*, *GmCCoAOMT10*, and *GmCCoAOMT12* showed almost no expression. *GmCCoAOMT1*, *GmCCoAOMT3*, *GmCCoAOMT4*, and *GmCCoAOMT5* also showed a changing characteristic of increasing first and then decreasing in the disease-resistant material JH, reaching the peak expression at 24 h, and there was no significant difference in expression in the susceptible material JL.

Further analysis of the expression patterns of candidate genes using the previous transcriptome FPKM data (**Supplementary Figure 3**; **Figure 4**) revealed that the transcriptional expression change trend was basically consistent with the qRT-PCR results, confirming the authenticity and reliability of the differential expression of candidate genes under stress in *P. manshurica*.

**Figure 17:**
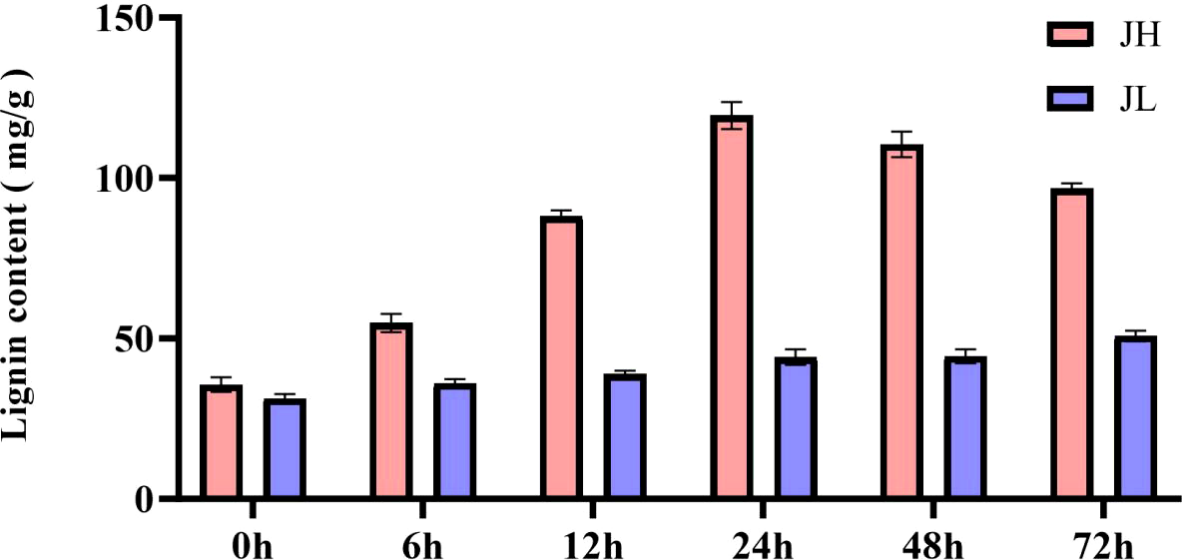
Changes in lignin content of JH and JL under *P. manshurica* infection. The red bar graph represents the high resistance to downy mildew JH, and the blue bar graph represents the high susceptibility to downy mildew JL.

To provide evidence from a physiological perspective for the functional correlation of the candidate genes, the lignin content in leaves at different time points after inoculation of *P. manshurica* with JH and JL was simultaneously measured (**Supplementary Table 9**; **Figure 17**). The results showed that the lignin accumulation trend in the disease-resistant material JH was highly consistent with the expression pattern of the candidate genes: it continued to rise at 6, 12, and 24 h, reached the peak at 24 h, and slightly declined at 48 and 72 h, and the lignin content at each time point was significantly higher than that of the 0-h control. Meanwhile, in the susceptible material JL, the lignin content showed no significant fluctuation throughout the infection period and was significantly lower than JH at the same time points.

The results of the comprehensive pathway analysis, gene expression, and physiological indicators show that *GmCAD14*, *GmCAD26*, *GmCAD28*, *GmCAD29*, and *GmCAD36* as well as *GmCCoAOMT1*, *GmCCoAOMT3*, *GmCCoAOMT4*, and *GmCCoAOMT5* can respond to soybean downy mildew infection and belong to potential candidate genes for resistance. *P. manshurica* infection can induce significant upregulation of these genes in resistant soybeans, thereby promoting lignin synthesis and accumulation and enhancing the cell wall lignification level to improve plant resistance.

## 4 Discussion

CAD and CCoAOMT gene families have been identified in *Arabidopsis* (Kim et al., 2004), rice (Park et al., 2018), wheat (Yang et al., 2021), and other different species. Their functions involve plant growth and development, hormone signal transduction, stress response, and other aspects. However, research on CAD and CCoAOMT gene families in soybean has not been reported. In this study, the members of *GmCAD* and *GmCCoAOMT* gene families in soybean were identified by bioinformatics analysis, and the physical and chemical properties, chromosome localization, gene structure, and conserved motifs were analyzed. At the same time, the evolutionary relationship, cis-acting elements, and expression patterns in tissues were analyzed, and the expression patterns under the infection of *P. manshurica* were analyzed in order to lay a foundation for the further study of the functional regulation mechanism of *GmCAD* and *GmCCoAOMT* gene families in soybean.

In this study, 41 *GmCAD* and 13 *GmCCoAOMT* genes were identified in soybean. Chromosomal distribution analysis showed that *GmCAD* genes were widely distributed on 19 chromosomes of soybean genome, while *GmCCoAOMT* genes were distributed on seven chromosomes of soybean genome. This feature is usually related to the amplification pattern and evolutionary history of gene families. The scattered distribution of gene families on chromosomes is mostly due to genomic fragment replication or genome-wide replication events (Cannon et al., 2004; Panchy et al., 2016), such as when the soybean genome has undergone multiple genome-wide replication events, which often lead to the dispersion and retention of genes on different chromosomes (Schmutz et al., 2010). This is consistent with the chromosome distribution characteristics of *GmCAD* and *GmCCoAOMT* in this study.

The physicochemical properties analysis showed that most *GmCAD* proteins were hydrophobic proteins, while *GmCCoAOMT* proteins were mainly hydrophilic proteins, which may be related to their different enzymatic properties and substrate binding properties (Sibout et al., 2005; Barros et al., 2015). Subcellular localization prediction showed that both family members were mainly located in the cytoplasm. It is worth noting that some members were predicted to be located in other organelles. This feature not only conforms to its core functional positioning in the lignin synthesis pathway but also suggests that the two types of genes have functional diversity.

In this study, the same subfamily members in gene structure and conserved motif analysis have similar exon–intron structure and conserved motif combinations, which is consistent with the analysis results of cinnamyl alcohol dehydrogenase family members in *Populus tomentosa* (Chao et al., 2014). Promoter cis-acting element analysis showed that *GmCAD* and *GmCCoAOMT* family members were rich in light response, hormone response, and stress response elements, indicating that the expression of *GmCAD* and *GmCCoAOMT* genes was synergistically regulated by developmental signals and environmental stress, which made them play a key and flexible hub role in plant growth and development, stress resistance and defense, and dynamic adaptation of lignin synthesis.

At the level of post-transcriptional regulation and protein interaction, the two gene families of *GmCAD* and *GmCCoAOMT* showed obvious differences in regulatory patterns and interaction networks. miRNAs are widely present in eukaryotes and regulate gene expression at the post-transcriptional level, which plays an important role in regulating the growth and development of plants (Lian et al., 2018). In this study, miRNA prediction analysis found that a large number of miRNAs targeted the regulation of *GmCAD* and *GmCCoAOMT* genes, and the regulation mode was complex, with the phenomenon of one-to-many and many-to-one, such as gma-miR172h-5p, gma-miR172i-5p, and gma-miR172j respectively targeting eight GmCAD genes, which is consistent with the results in oil palm (Zhou et al., 2024) and wheat (Zhang et al., 2022). It also further illustrates that miRNA plays a key role in the post-transcriptional regulation of soybean, which provides an important miRNA target reference for the subsequent analysis of the molecular regulation network of soybean lignin metabolism and molecular breeding improvement.

Co-expression network analysis showed that there was a wide range of co-expression interactions within the *GmCAD* gene. *GmCAD17* and *GmCAD35*, as the core nodes, interacted with many family members, suggesting that they may act as hubs to coordinate the co-expression or functional modules within the *GmCAD* family. In contrast, there is no co-expression interaction between members of the *GmCCoAOMT* family, and there is no interaction between the two families. These results suggest that *GmCAD* and *GmCCoAOMT* may be relatively independent in the formation of transcriptional regulatory networks and potential protein complexes, although *GmCAD* and *GmCCoAOMT* are connected upstream and downstream in the lignin synthesis pathway. *GmCAD* gene family members may optimize metabolic flux by forming multi-enzyme complexes or establishing close regulatory synergy networks, while the regulation of *GmCCoAOMT* may be more likely to rely on independent transcriptional or post-transcriptional mechanisms or form specific interactions with other non-family proteins to achieve functional regulation (Li Y. K et al., 2022; Wu et al., 2024).

At the level of phylogenetic evolution, the three subgroups of the *GmCAD* gene family showed obvious species aggregation characteristics. The 24 members of subgroup I are all soybean *GmCAD* genes, and subgroup II only contains *GmCAD26* and *GmCAD34*, while subgroup III contains cross-species members of soybean, *Arabidopsis*, and rice. This grouping pattern is similar to the evolutionary characteristics of *GmCAD* gene family in *Gossypium hirsutum*. The *GmCAD* genes in *Gossypium hirsutum* can also be divided into species-specific subgroups and cross-species conserved subgroups (Zhang et al., 2024). It is speculated that this feature may be related to the adaptive differentiation of leguminous plants to specific physiological functions such as nitrogen fixation and lignin synthesis during evolution. In contrast, in the five subgroups of the *GmCCoAOMT* gene family, subgroups I, II, III, and V contained *Arabidopsis* genes, and only subgroup IV was unique to rice, which directly proved that the soybean *GmCCoAOMT* family was more closely related to the evolution of the dicotyledonous plant *Arabidopsis*, which was consistent with the result that the tea plant *CsCCoAOMT* family was more likely to form an evolutionary cluster with dicotyledonous plants (Lin et al., 2021). It was further confirmed that the *GmCCoAOMT* gene may maintain the core role of methyltransferase in lignin synthesis through the retention of conserved functional modules after the differentiation of monocotyledonous and dicotyledonous plants.

The analysis of collinearity and selection pressure further revealed the evolutionary stability of the gene family. The Ka/Ks values of 12 pairs of homologous genes in 13 pairs of homologous genes of the *GmCAD* gene were less than 1, and the Ka/Ks values of nine pairs of homologous genes of the *GmCCoAOMT* gene were less than 1. The homologous gene separation time of *GmCAD* and *GmCCoAOMT* covered 6.25–251.88 and 9.95–128.54 Mya, respectively. This is highly consistent with the evolutionary trend of *Solanum tuberosum StCCoAOMT* gene family based on purification selection (Peng et al., 2024). There are 14 pairs of collinearity genes between soybean *GmCAD* and *Arabidopsis* genome and six pairs of collinearity genes between soybean *GmCCoAOMT* and *Arabidopsis* genome, which are much higher than the collinearity genes between soybean and rice (four pairs of *GmCAD*, no pairs of *GmCCoAOMT*). This result indicates that soybean and *Arabidopsis* have higher conservation in the evolution of *GmCAD* and *GmCCoAOMT* genes, and their genetic relationship is closer than that of soybean and rice.

The soybean *GmCAD* and *GmCCoAOMT* family members showed obvious tissue-specific expression patterns—for example, *GmCAD27* was highly expressed in seeds at 35 DAF and 42 DAF but lowly expressed in tissues such as roots and nodules. *GmCAD30* was highly expressed in pod shells at 14 DAF but lowly expressed in young leaves, roots, and nodules, whereas *GmCCoAOMT13* was highly expressed in young leaves, flowers, and 1-cm pods but showed low expression levels in seeds at 42 DAF. Meanwhile, members that were not expressed in any of the examined tissues were present in both families (e.g., *GmCAD16*, *GmCCoAOMT7*, and *GmCCoAOMT10*), suggesting that they may be activated only at specific developmental stages or under stress conditions. This highly differentiated expression pattern is closely related to the complexity and specificity of lignin biosynthesis pathways, consistent with the *PgCAD* gene family in pomegranate (*Punica granatum*) (Hu et al., 2022).

In the expression analysis under the infection of *P. manshurica*, the expression levels of *GmCAD14*, *GmCAD26*, *GmCAD28*, *GmCAD36*, *GmCCoAOMT1*, *GmCCoAOMT3*, *GmCCoAOMT4*, and *GmCCoAOMT5* in the HR accession JH showed a trend of increasing first and then decreasing after inoculation with *P. manshurica*, and these peaked at 24 h. This expression pattern is consistent with the results of transcriptome data, and these genes may be involved in the early resistance defense response of soybean. As a key component of plant cell wall, lignin is an important mechanism to form a physical barrier to prevent the invasion and spread of pathogenic fungus (Miedes et al., 2014). Combined with the key role of CAD and CCoAOMT genes in lignin synthesis, it is speculated that these genes may form a physical barrier by regulating lignin accumulation, thereby enhancing the resistance of soybean to downy mildew.

In summary, this study identified and deeply analyzed the *GmCAD* and *GmCCoAOMT* gene families in soybean for the first time through systematic bioinformatics analysis and revealed their distribution characteristics on chromosomes, physical and chemical properties, gene structure, phylogenetic relationship, expression regulation network, and expression patterns in response to *P. manshurica* stress. The results showed that the two gene families had undergone a complex evolutionary process in soybean, formed a diverse membership and expression regulation pattern, and showed high evolutionary conservation with dicotyledonous plants such as *Arabidopsis*. Especially under the condition of *P. manshurica* infection, multiple *GmCAD* and *GmCCoAOMT* genes showed significant induced expression patterns, suggesting that they may play a key role in the defense response of soybean. This study provides important theoretical basis and data support for further elucidating the biological functions of *GmCAD* and *GmCCoAOMT* genes in soybean lignin synthesis, growth and development, and stress response and also provides potential gene resources and regulatory targets for soybean resistance molecular breeding.

Finally, it is important to acknowledge two key limitations of the present study. First, the observation that defense-related gene expression and lignin content peak at 24 h in the resistant genotype JH reflects a correlative response to infection, rather than a definitive proof that this specific response directly stops *P. manshurica* colonization. While the coordinated upregulation of lignin biosynthetic genes and concomitant lignin accumulation strongly support a plausible functional link to resistance, establishing direct causality will require future genetic validation (e.g., overexpression or knockout of key pathway genes). Second, our identification of miRNA targets and promoter cis-elements is based entirely on *in silico* predictions. Although these analyses provide valuable preliminary insights into potential post-transcriptional and transcriptional regulation of lignin pathway genes, they lack direct biological confirmation. Future work using dual-luciferase reporter assays, RNA immunoprecipitation, or electrophoretic mobility shift assays will be necessary to verify these predicted interactions.

## 5 Conclusions

In this study, 41 *GmCAD* genes and 13 *GmCCoAOMT* genes were identified in the soybean genome for the first time, and their physicochemical properties, gene structure, conserved motifs, and chromosome distribution characteristics were systematically analyzed. Phylogenetic analysis showed that both gene families underwent purification selection (Ka/Ks < 1), and the evolutionary correlation with *Arabidopsis* was significantly stronger than that with rice, reflecting functional conservation and species-specific differentiation. Promoter cis-acting elements and miRNA regulatory network analysis showed that the two types of genes contained abundant stress response elements and were regulated by a variety of miRNA post-transcriptional regulation, which provided important clues for their functional regulation mechanism. Tissue expression pattern analysis confirmed that the *GmCAD* and *GmCCoAOMT* genes had obvious tissue specificity, while *GmCAD14*, *GmCAD26*, *GmCAD28*, *GmCAD29*, *GmCAD36*, *GmCCoAOMT1*, *GmCCoAOMT3*, *GmCCoAOMT4*, and *GmCCoAOMT5* may be candidate genes for soybean downy mildew resistance under *P. manshurica* infection. This study systematically elucidated the core characteristics and disease-resistance-related functions of soybean CAD and CCoAOMT gene families, which provided a theoretical basis for in-depth analysis of the molecular mechanism of lignin synthesis and provided excellent gene resources and key target support for molecular breeding of soybean resistance to downy mildew.

## Data Availability

The datasets presented in this study can be found in online repositories. The names of the repository/repositories and accession number(s) can be found in the article/**Supplementary Material**.
