# Supplementary material for: Genome-wide identification of CAD and CCoAOMT gene families in soybean and analysis of expression patterns under Peronospora manshurica (P. manshurica) infection
Source: Front Plant Sci. 2026 Jun 16;17:1834683. doi: 10.3389/fpls.2026.1834683 (PMC13314508; doi:10.3389/fpls.2026.1834683)
Supplement: Supplementary file 1 [file DataSheet1.zip › Supplementary figures+tables/Supplementary figures/Supplementary figures.docx]

**Supplementary figures**


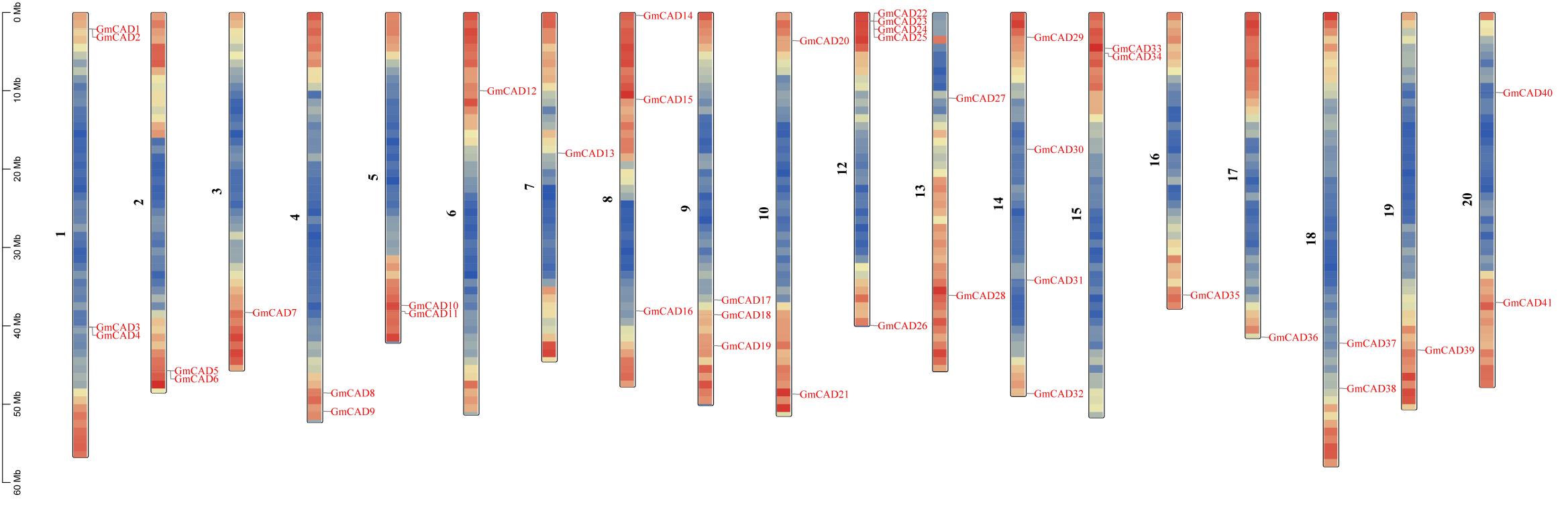


**Supplementary Figure 1** The chromosomal location of the *GmCAD* gene in soybeans. On the left side of each bar chart, the numbers represent chromosomes. Different colors on each chromosome indicate the distribution of gene density


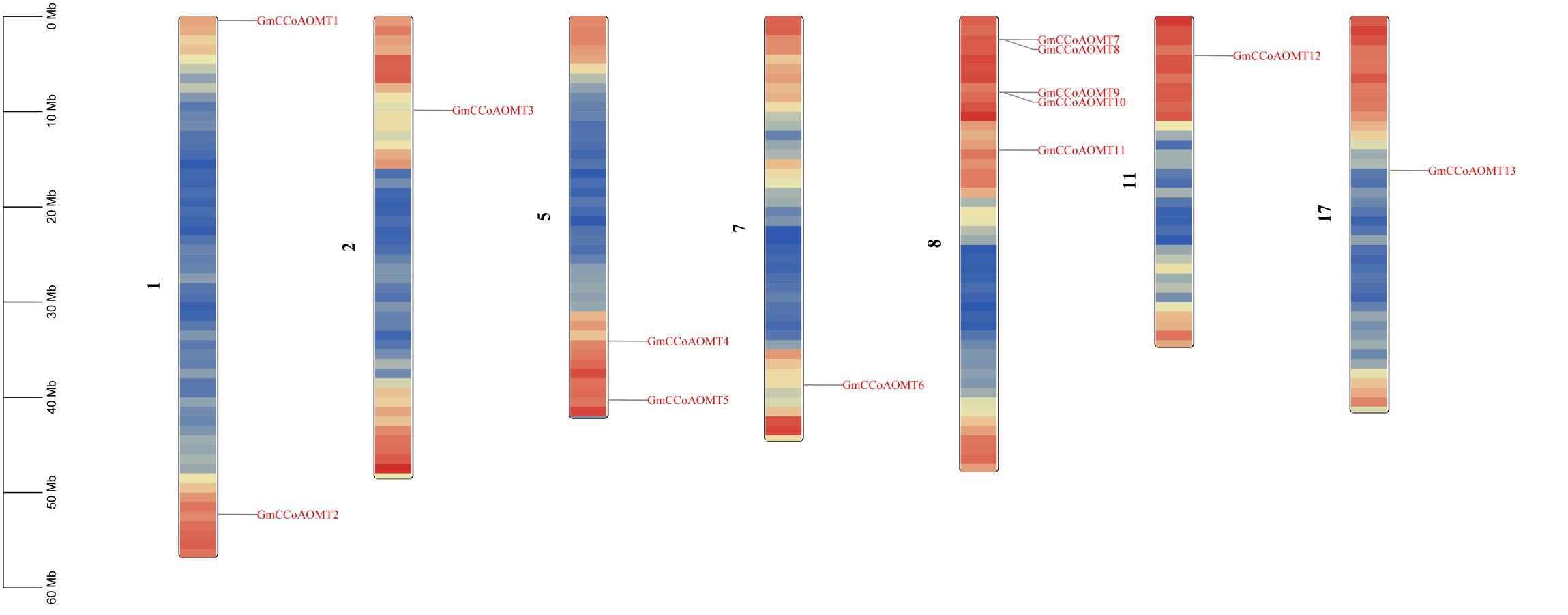


**Supplementary Figure 2** The chromosomal location of the *GmCCoAOMT* gene in soybeans. On the left side of each bar chart, the numbers represent chromosomes. Different colors on each chromosome indicate the distribution of gene density


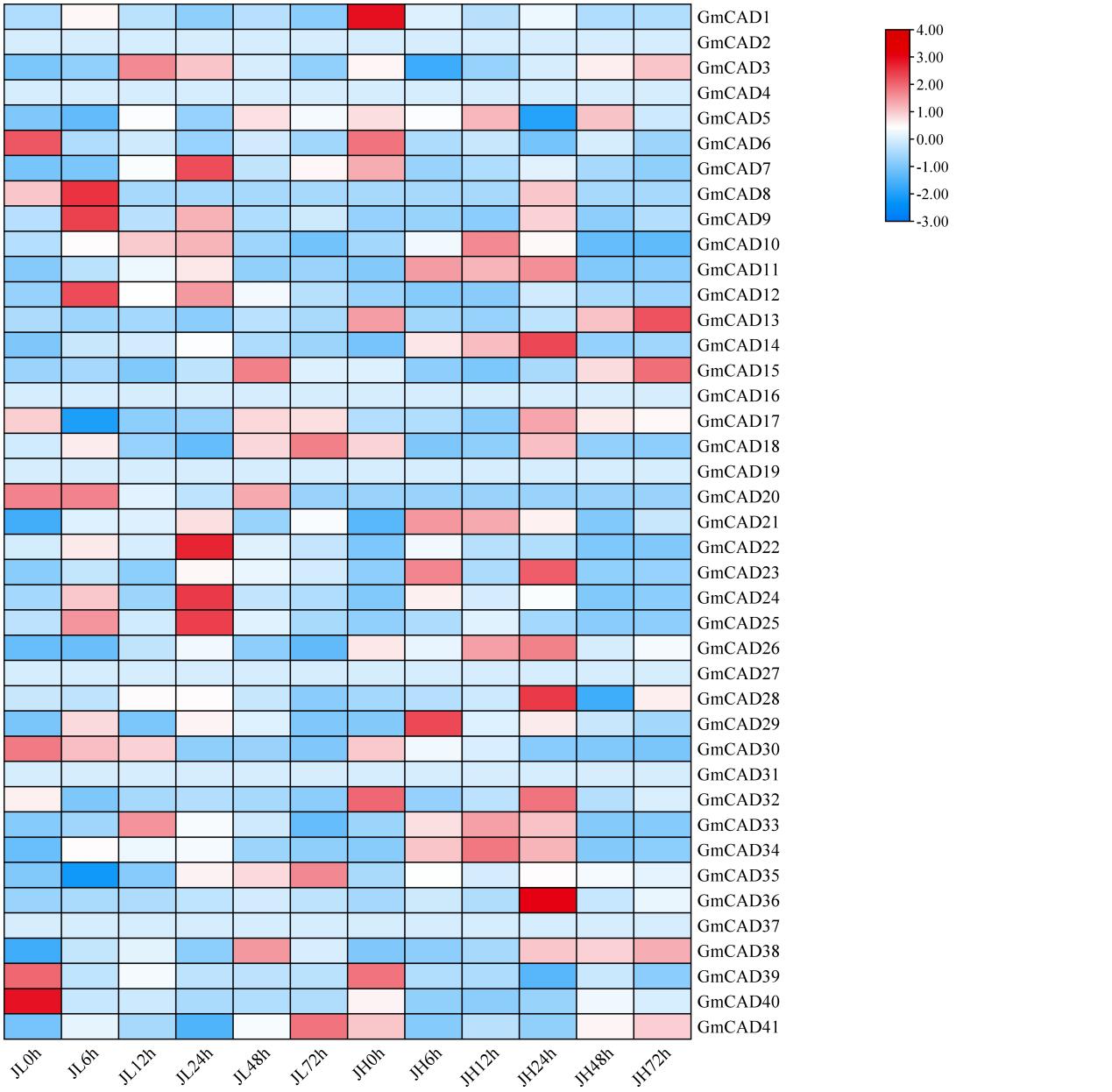


**
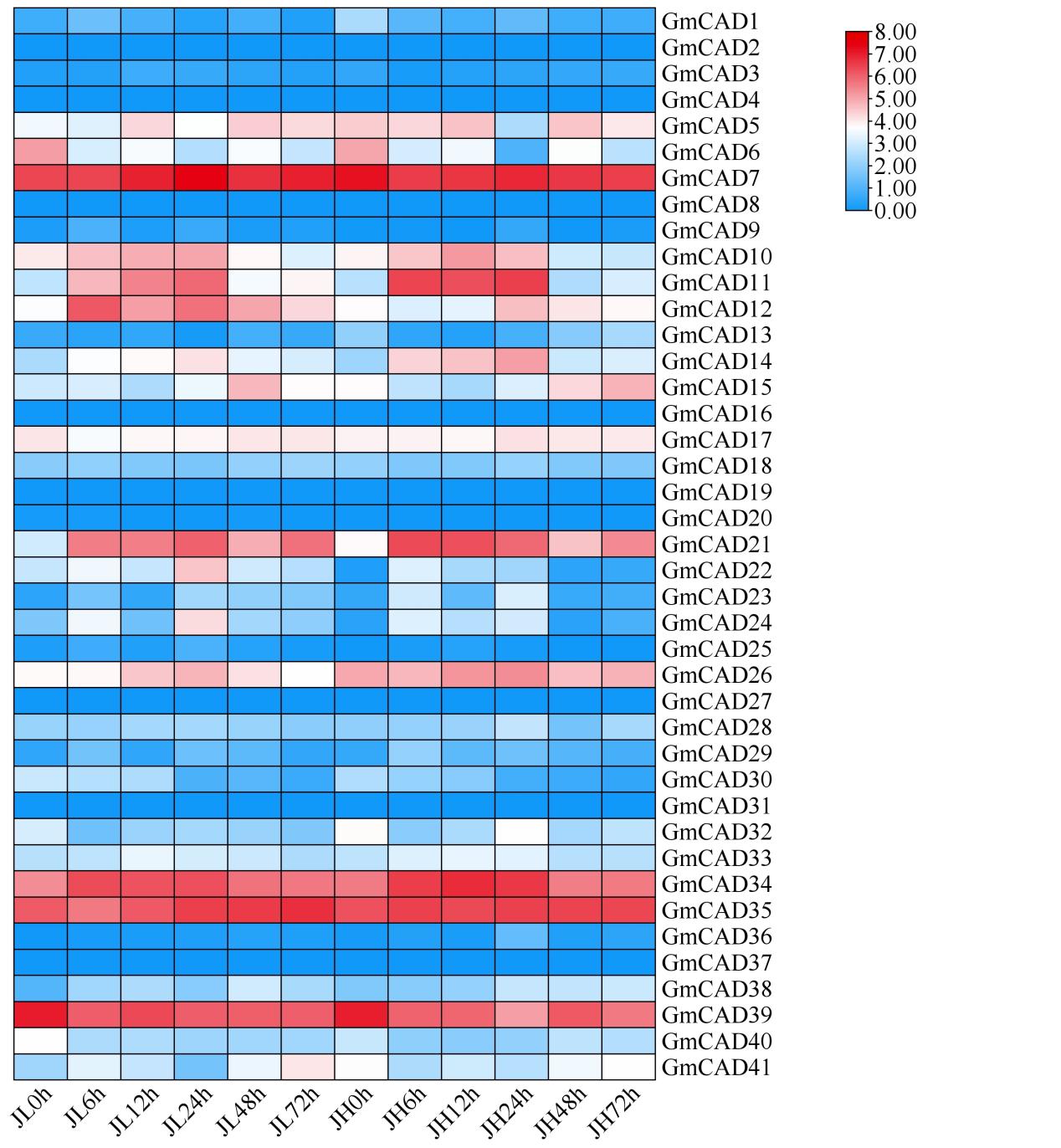
**

**Supplementary Figure 3** Expression pattern of the *GmCAD* gene in soybean under infection by *P. manshurica*. The expression values are plotted using log2(FPKM + 1). From blue (low expression) to red (high expression)


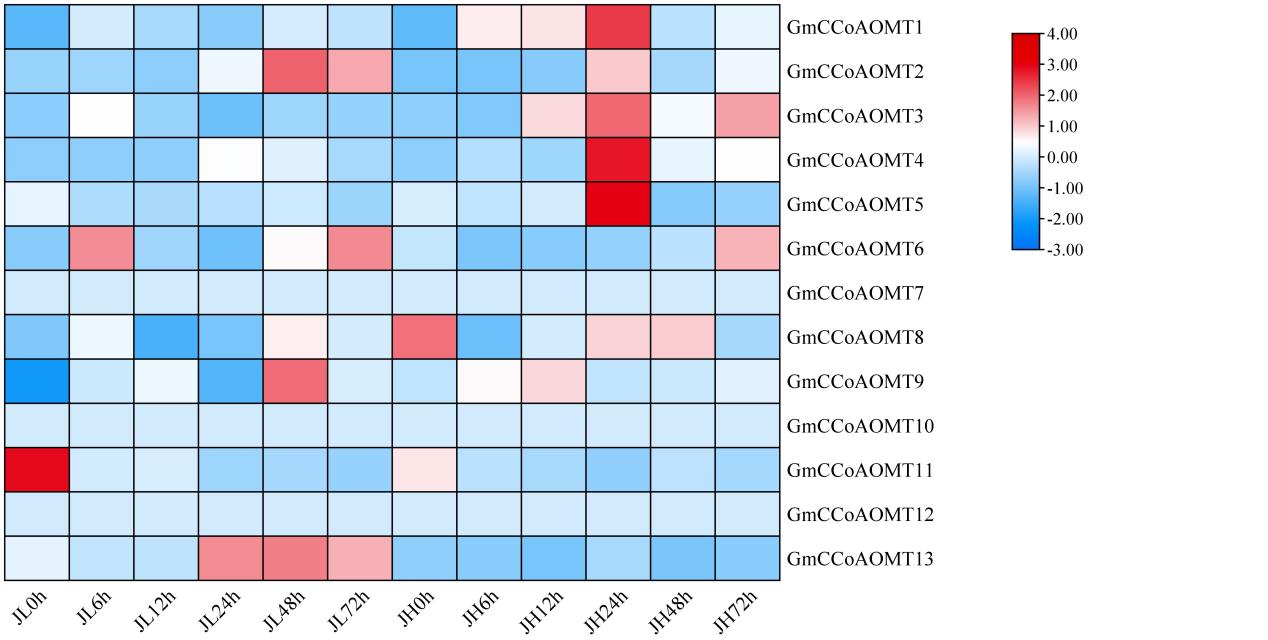


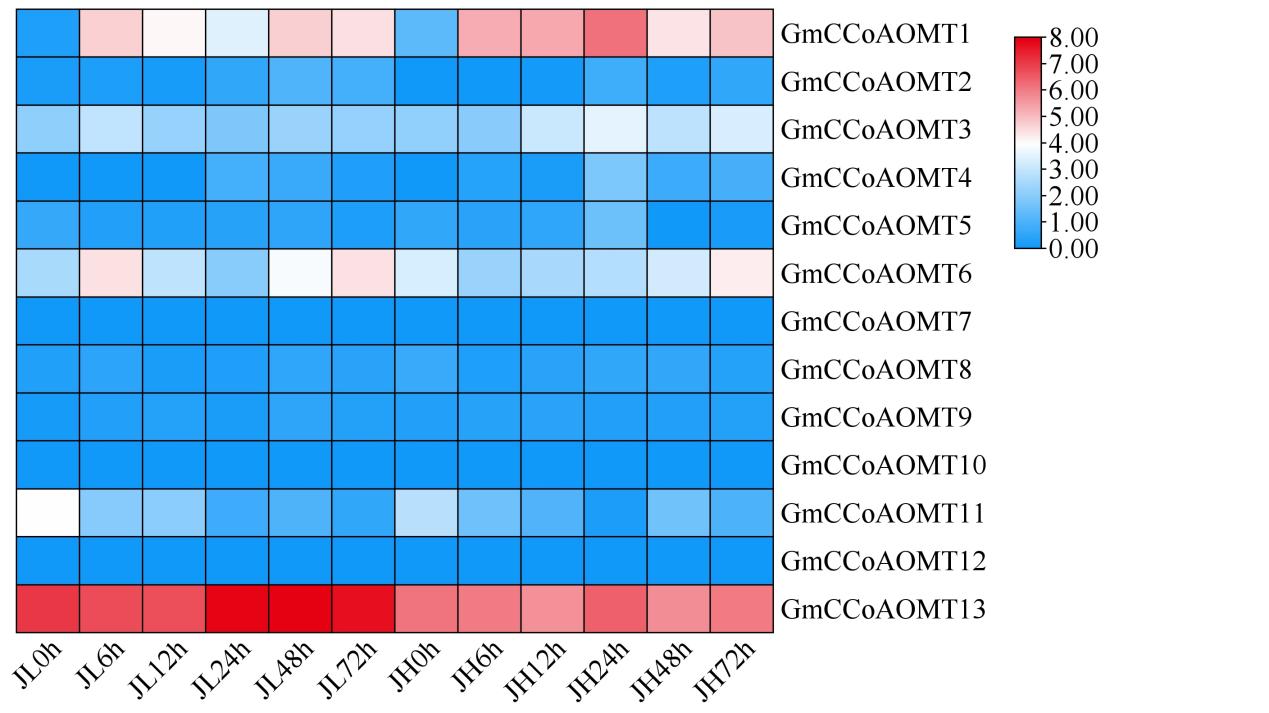


**Supplementary Figure 4** Expression pattern of the *GmCCoAOMT* gene in soybean under infection by *P. manshurica* (FPKM). The expression values are plotted using log2(FPKM + 1). From blue (low expression) to red (high expression)
